# SosA inhibits cell division in *Staphylococcus aureus* in response to DNA damage

**DOI:** 10.1111/mmi.14350

**Published:** 2019-08-16

**Authors:** Martin S. Bojer, Katarzyna Wacnik, Peter Kjelgaard, Clement Gallay, Amy L. Bottomley, Marianne T. Cohn, Gunnar Lindahl, Dorte Frees, Jan‐Willem Veening, Simon J. Foster, Hanne Ingmer

**Affiliations:** ^1^ Department of Veterinary and Animal Sciences, Faculty of Health and Medical Sciences University of Copenhagen Copenhagen Denmark; ^2^ Centre for Bacterial Stress Response and Persistence University of Copenhagen Copenhagen Denmark; ^3^ Department of Molecular Biology and Biotechnology, The Krebs Institute University of Sheffield Sheffield UK; ^4^ Department of Fundamental Microbiology University of Lausanne Lausanne Switzerland; ^5^ Present address: The ithree Institute University of Technology Sydney Sydney NSW Australia

## Abstract

Inhibition of cell division is critical for viability under DNA‐damaging conditions. DNA damage induces the SOS response that in bacteria inhibits cell division while repairs are being made. In coccoids, such as the human pathogen, *Staphylococcus aureus*, this process remains poorly studied. Here, we identify SosA as the staphylococcal SOS‐induced cell division inhibitor. Overproduction of SosA inhibits cell division, while *sosA* inactivation sensitizes cells to genotoxic stress. SosA is a small, predicted membrane protein with an extracellular C‐terminal domain in which point mutation of residues that are conserved in staphylococci and major truncations abolished the inhibitory activity. In contrast, a minor truncation led to SosA accumulation and a strong cell division inhibitory activity, phenotypically similar to expression of wild‐type SosA in a CtpA membrane protease mutant. This suggests that the extracellular C‐terminus of SosA is required both for cell division inhibition and for turnover of the protein. Microscopy analysis revealed that SosA halts cell division and synchronizes the cell population at a point where division proteins such as FtsZ and EzrA are localized at midcell, and the septum formation is initiated but unable to progress to closure. Thus, our findings show that SosA is central in cell division regulation in staphylococci.

## Graphical abstract

*Staphylococcus aureus* is a human pathogen and a model organism for cell division in spherical bacteria. We show that SosA is the DNA‐damage‐inducible cell‐division inhibitor in *S. aureus* responsible for cessation of the cell cycle prior to division plate completion. The extracellular domain of SosA appears essential for activity but is also a likely target for regulation by the CtpA protease. This represents the first description of a cell division inhibition process in coccoid bacteria.

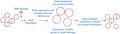

## Introduction

Bacteria multiply by coordinated and essential DNA replication and cell division events, two important biological processes that are valuable targets for antimicrobial therapy (Lock and Harry, 2008; Adams and Errington, 2009; Robinson *et al.*, 2012; Sass and Brötz‐Oesterhelt, 2013; den Blaauwen *et al.*, 2017). In the event of DNA damage, the SOS response is activated and ensures that cell division is delayed until the DNA is repaired. The SOS regulon is controlled by the conserved LexA repressor, which is inactivated in response to RecA, a sensor of DNA damage at stalled replication forks, binding to single‐stranded DNA (Kelley, 2006; Kreuzer, 2013; Baharoglu and Mazel, 2014). The SOS response has mostly been studied in *Escherichia coli*, a Gram‐negative rod‐shaped bacterium where the LexA‐regulated gene, *sulA*, encodes a cell division inhibitor. This inhibitor suppresses division by binding to FtsZ, which acts as a scaffold for the assembly of cell division components at the division site (Huisman and D'Ari, 1981; Huisman *et al.*, 1984; Jones and Holland, 1985; Higashitani *et al.*, 1995; Mukherjee *et al.*, 1998; Cordell *et al.*, 2003; Adams and Errington, 2009). In rod‐shaped bacteria, inhibition of cell division leads to filamentation as a consequence of lateral peptidoglycan synthesis. This phenotype is also observed in *E. coli* mutants lacking the Lon protease of which SulA is a substrate, further substantiating the role of SulA in cell division inhibition and filamentation (Mizusawa and Gottesman, 1983; Schoemaker *et al.*, 1984).

SulA is poorly conserved in species outside Enterobacteriaceae. The α‐proteobacterium *Caulobacter crescentus* encodes an SOS‐induced cell division inhibitor SidA that does not show homology to SulA. In contrast to cytosolic SulA, SidA is a membrane‐anchored, small protein that does not interact with FtsZ but rather with later cell division proteins (Modell *et al.*, 2011). In this species, yet another, possibly redundant, cell division inhibitor DidA is implicated in survival during DNA damage, while being independent of the SOS response (Modell *et al.*, 2014). Hence, it appears that bacteria choose fundamentally different ways to orchestrate regulated cell division inhibition in response to DNA damage. For Gram‐positive, spherical cells such as *Staphylococcus aureus*, however, little is known of how SOS induction is coupled to cell division nor have specific posttranslational mechanisms for the negative regulation of cell division inhibition been identified.

**Figure 1:**
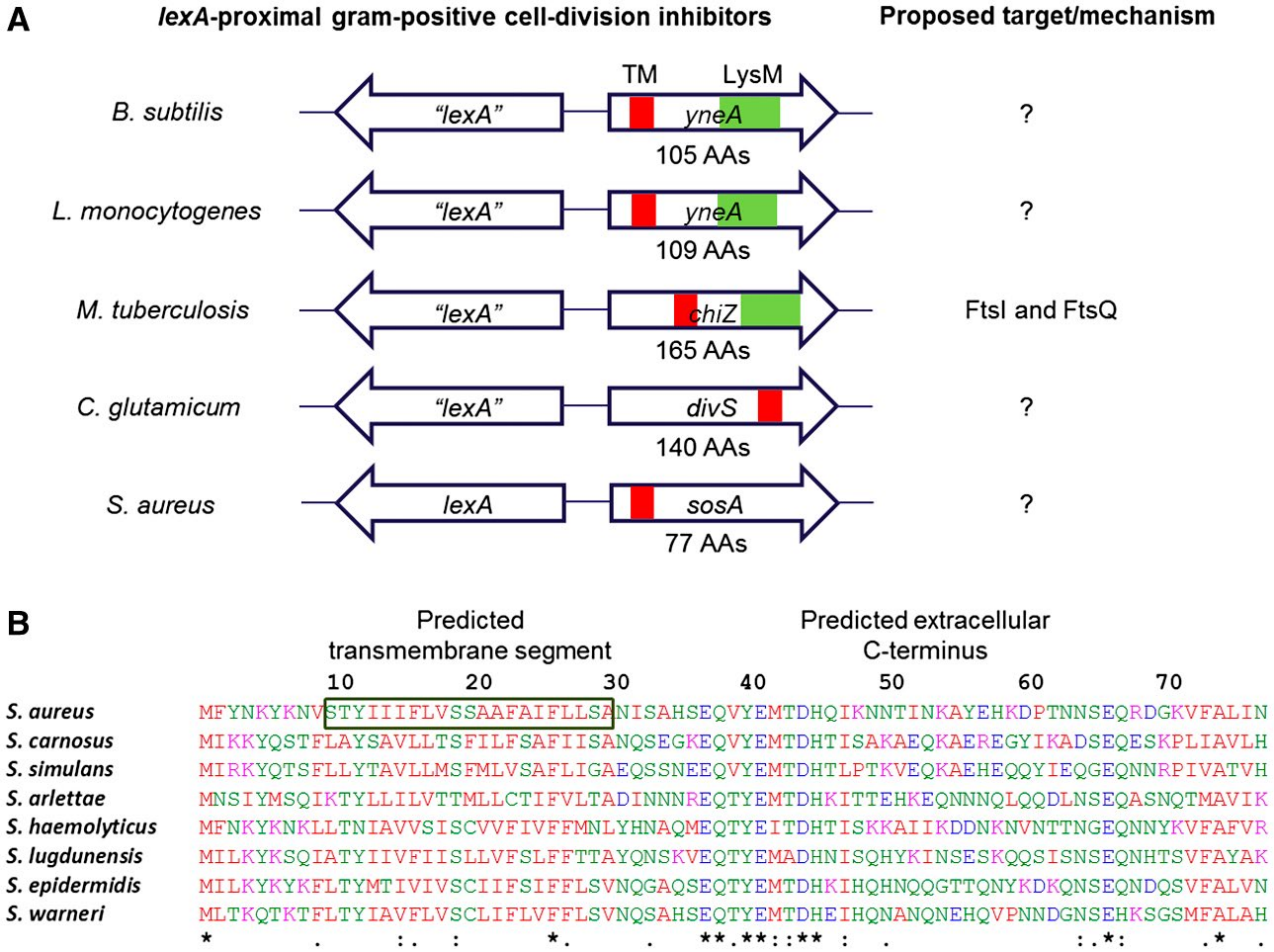
Gram‐positive SOS‐controlled cell division inhibitors. A. Schematic representation of genes encoding characterized Gram‐positive SOS‐regulated cell division inhibitors (not drawn to scale) including the uncharacterized *sosA* from *S. aureus*. Despite considerable sequence divergence, these genes are commonly chromosomally co‐localized with *lexA* homologous genes. The cell division inhibitors carry a single transmembrane domain (TM), and several proteins have an additional LysM domain. B. Alignment (CLUSTAL O[1.2.4]) of SosA sequences deduced from open reading frames next to *lexA* in *S. aureus* strain 8325‐4 (YP_499864) and seven *Staphylococcus* species: *S. carnosus* (CAL27889), *S. simulans* (AMG96201), *S. arlettae* (EJY94737), *S. haemolyticus* (YP_253482), *S. lugdunensis* (YP_003471776), *S. epidermidis* (YP_188489) and *S. warneri* (EEQ79882). The proteins are 77 amino acids long and are characterized by a predicted transmembrane segment at AAs 10–30 (for *S. aureus* SosA) and a predicted extracellular C‐terminal (TOPCONS [Tsirigos et al., 2015]) with considerable sequence conservation at the membrane‐proximal portion (‘*’ indicates fully conserved residues, ‘:’ indicates conservation of residues with highly similar properties).

*S. aureus* is a serious Gram‐positive human pathogen, notorious for being implicated in a wide range of infections and for being able to acquire resistance toward important antibiotic classes. It originally received its name from the grape‐like clusters of coccoid cells that result from the unique cell division process which occurs in three consecutive orthogonal planes (Tzagoloff and Novick, 1977; Turner *et al.*, 2010; Pinho *et al.*, 2013). In this organism, we and others have previously identified *lexA* and noted an open reading frame (designated *sosA*) that is divergently transcribed from *lexA* and controlled by the LexA repressor and the SOS response (Anderson *et al.*, 2006; Cirz *et al.*, 2007; Mesak *et al.*, 2008; Cohn *et al.*, 2011). The location of *sosA* adjacent to *lexA* indicated that it might encode a cell division inhibitor, as similar gene synteny has been observed for SOS‐induced cell division inhibitors encoded by Gram‐positive, rod‐shaped bacteria, namely *Bacillus subtilis* (Kawai *et al.*, 2003), *Bacillus megaterium* (Buchholz *et al.*, 2013), *Listeria monocytogenes* (van der Veen *et al.*, 2007; 2010), *Mycobacterium tuberculosis* (Chauhan *et al.*, 2006) and *Corynebacterium glutamicum* (Ogino *et al.*, 2008) (Fig. 1A). The cell division inhibitors of these organisms display no similarity to SulA, and within the group they generally show little homology with the exceptions of YneA from *L. monocytogenes* and ChiZ from *M. tuberculosis* (Chauhan *et al.*, 2006) that resemble *B. subtilis* YneA. Commonly, however, they have a single membrane spanning segment, a predicted extracellular C‐terminus and, except for DivS from *C. glutamicum*, a LysM domain, which is a ubiquitous peptidoglycan‐binding motif (Buist *et al.*, 2008; Mesnage *et al.*, 2014), suggesting that membrane and/or cell wall localization is a common feature characterizing Gram‐positive cell division inhibitors. In this line, it is interesting that *M. tuberculosis* ChiZ was reported to have cell wall hydrolase activity (Chauhan *et al.*, 2006), which could be functionally important for LysM‐containing cell division inhibitors.

The fundamental processes of staphylococcal cell division have been studied spatiotemporally using super‐resolution microscopy techniques (Turner *et al.*, 2010; Monteiro *et al.*, 2015), and recent efforts combining genetic approaches have unveiled the molecular mechanism in unprecedented detail (Lund *et al.*, 2018; Monteiro *et al.*, 2018). Here, we demonstrate that *sosA* encodes the SOS‐inducible cell division inhibitor in *S. aureus* and document its impact on cell division following treatment with DNA damaging agents. Moreover, we identify a possible mechanism for the proteolytic control of endogenous cell division inhibition. Thus, we further our insight into these basic biological phenomena, which could lead to the development of new antimicrobials targeting the cell division machinery in *S. aureus*.

## Results

### Conservation of *sosA* in staphylococci

In *S. aureus*, the open reading frame located adjacent to *lexA* (divergently transcribed) was named *sosA* and hypothesized to encode an inhibitor of cell division (Fig. 1A) (Cohn *et al.*, 2011). The 77‐amino‐acid‐long product has homology and 25%–60% amino acid (AA) identity to proteins encoded by genes occupying the same chromosomal location in a range of staphylococci (Figs 1B and S1), while showing no clear homology to the known Gram‐positive cell division inhibitors. Of note, the C‐terminal part of SosA lacks a LysM domain, which is present in the cell division inhibitors YneA and ChiZ (Mo and Burkholder, 2010; Vadrevu *et al.*, 2011). The N‐terminal half of SosA includes a putative transmembrane (TM) domain, and the C‐terminal part is predicted to be located extracellularly (TOPCONS server [Tsirigos *et al.*, 2015]). Among the different staphylococcal species, a highly conserved sequence is located just C‐terminally of the predicted TM domain and, although less striking, some conservation also seems to exist in the extreme C‐terminus (Fig. 1B).

### Genotoxic stress inhibits cell division in an SosA‐dependent manner

**Figure 2:**
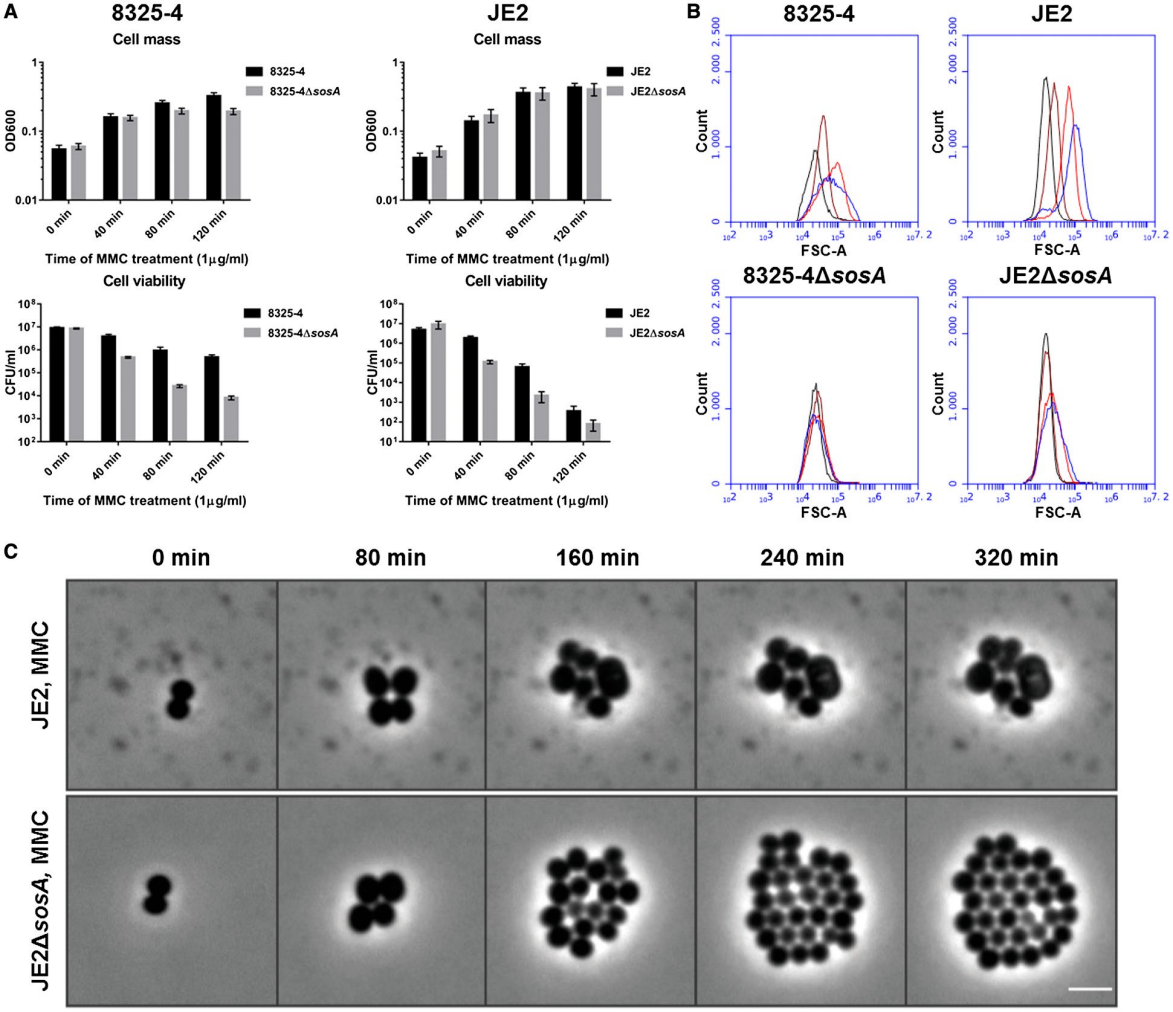
SosA supports survival of *S. aureus* subjected to lethal DNA damage and is involved in bacterial swelling. A. Culture optical density at 600 nm and cell viability of *S. aureus* strains 8325‐4 and JE2 in comparison with their respective *ΔsosA* mutants upon challenge with a lethal dose of mitomycin C (MMC, 1 µg ml^−1^) for 2 h. Error bars represent the standard deviation from three biological replicates. B. Cell size of 8325‐4 and JE2 WT and *ΔsosA* mutants exposed to mitomycin C estimated by flow cytometry (FSC‐A). Cells were grown exponentially prior to MMC addition at an OD_600_ of 0.05. Samples were taken after 0 (black), 40 (brown), 80 (red) and 120 (blue) min of incubation with MMC. C. Effect of MMC treatment (0.04 µg ml^−1^) on cell shape and cell number of JE2 WT and JE2Δ*sosA* as visualized by time‐lapse phase‐contrast microscopy. Scale bar represents 2 µm.

To analyze whether SosA serves as an SOS‐induced cell division inhibitor, we exposed 8325‐4, the common laboratory *S. aureus* strain, and JE2, a derivative of the clinically relevant USA300 lineage, to a lethal concentration of the DNA‐damaging agent Mitomycin C (MMC) and observed induction of SosA expression in both strains by western blot analysis (Fig. S2A). In contrast, no SosA was observed in deletion mutant derivatives but was expressed in a complemented strain (Fig. S2A). Upon exposure to the same lethal dose of MMC for a 2 h period, wild‐type (WT) cells showed 10–100‐fold greater survival than the mutant cells lacking SosA over the course of the experiment, whereas the optical densities of both cell‐type cultures were comparable (Fig. 2A). Although the killing kinetics of 8325‐4 and JE2 are different, the survival ratio of WT vs. mutant remained comparable, with WT showing 32‐ and 54‐fold greater survival at 80 min for the respective strain backgrounds. Flow cytometry measurements of cell size distributions revealed that treatment with MMC led to a three‐ and sixfold increase in forward light scatter (FSC‐A) values in 8325‐4 and JE2, respectively, while the cell size distribution of the mutants lacking *sosA* remained essentially constant (Fig. 2B). The constant cell size but increasing optical densities of the *sosA* mutant populations implies that during DNA damage these cells, in contrast to WT cells, continue to divide. Indeed, this striking phenotype was confirmed by time‐lapse microscopy, where WT cells increased in size over time, occasionally growing so large that lysis occurred, while the size of the mutant cells lacking *sosA* stayed the same and cell division continued (Fig. 2C and Movie S1). The phenotypes of the 8325‐4 *sosA* mutant were abrogated in a strain complemented with a chromosomal copy of *sosA* expressed from its native promoter (Fig. S2B and C). Thus, SosA seems to prevent or delay cell division; maintain viability of *S. aureus* under DNA damaging conditions, and as a consequence of continued activity of the peptidoglycan synthesis machinery cell size increases.

### SosA overexpression inhibits cell division

**Figure 3:**
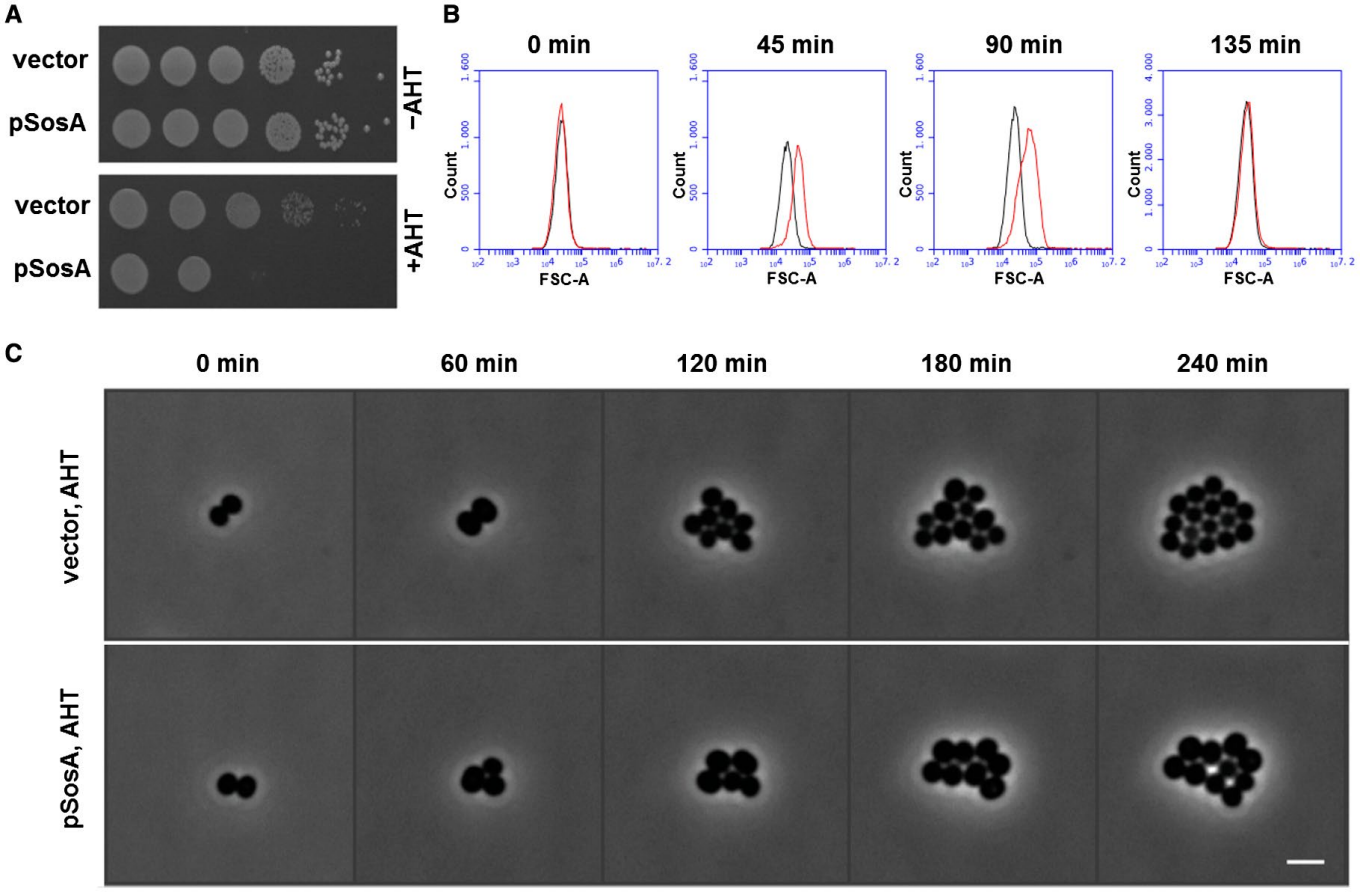
Expression of *sosA* alone interferes with *S. aureus* growth. A. The effect of a controlled expression of *sosA* on the ability to form colonies was assessed in *S. aureus* RN4220. Strains carrying either the vector control (vector) or *sosA* under an anhydrotetracycline (AHT)‐inducible promoter (pSosA) were grown exponentially to an OD_600_ of 0.5, serially 10‐fold diluted and plated on TSA plates in the presence or absence of TSA + 300 ng ml^−1^ of AHT. The plates were incubated overnight at 37°C and imaged. B. Evaluation of cell size distribution by flow cytometry (FSC‐A) of *S. aureus* RN4220 containing the control vector (black) or pSosA (red). Cells were grown exponentially prior to induction with 100 ng ml^−1^ of AHT. At the indicated time points, the cells were collected and analyzed by flow cytometry. C. Visualization of cell size increase and reduction of cell number of JE2/pRAB12‐*lacZ* (control) and JE2/pRAB12‐*sosA* in the presence of 200 ng ml^−1^ of AHT by time‐lapse phase‐contrast microscopy at 37°C. Scale bar represents 2 µm.

If SosA is indeed a cell division inhibitor in *S. aureus*, it can be predicted that overexpression of the protein should be sufficient to inhibit colony formation even under conditions with no DNA damage. Hence, we expressed *sosA* episomally from an anhydrotetracycline (AHT)‐inducible promoter and found that while the control vector did not affect cell viability, the plating efficiency of cells carrying the *sosA* overexpression plasmid was markedly compromised under inducing conditions (Fig. 3A). When grown in liquid culture and compared to the control, the SosA overexpressing cells showed a two to threefold increase in forward light scatter values, reflecting that the size of the *sosA*‐expressing cells increased (Fig. 3B). Microscopy observations over time confirmed the slight increase in cell size and the reduction in the overall number of cells upon SosA overproduction (Fig. 3C and Movie S2). Interestingly, contrary to what was observed under DNA‐damaging conditions (Fig. 2B), we found that ectopic induction of SosA caused only a transient increase in cell size (compare time points 45 and 90–135 min in Fig. 3B), indicating the existence of a negative regulatory mechanism.

### The C‐terminal part of SosA is functionally essential and possibly required for autoregulation

**Figure 4:**
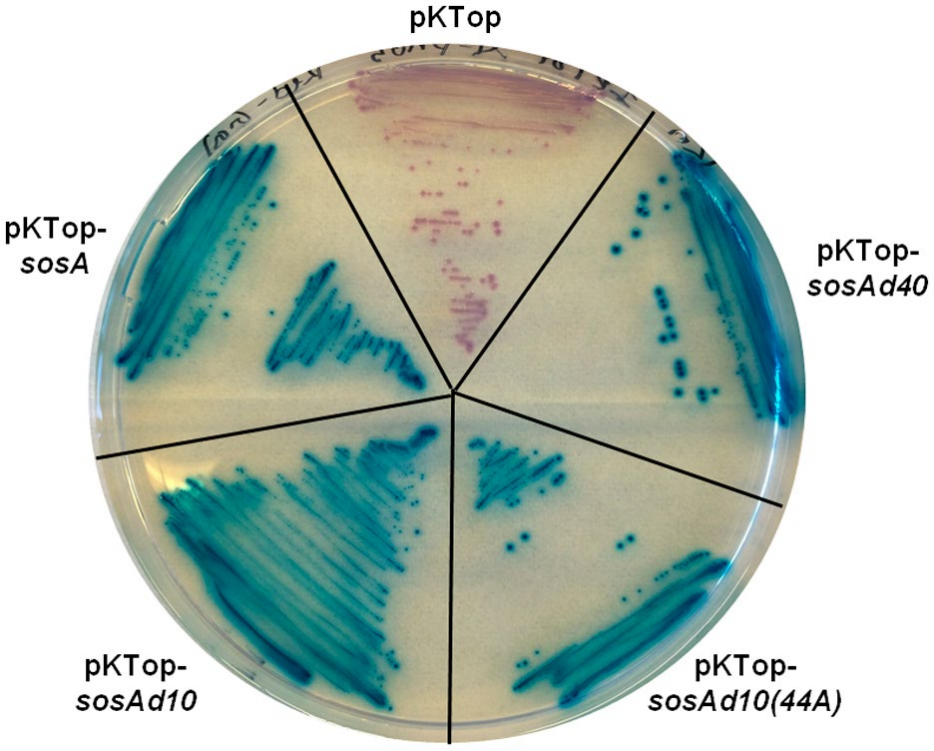
Evaluation of membrane localization of SosA and truncated variants SosAd10, SosAd10(44A) and SosAd40 by in frame fusion at the C‐terminus to PhoA‐LacZ in pKTop (the truncated variants are explained in Figs 5 and 6). *E. coli* IM08B cells carrying the constructs were streaked on a dual‐indicator plate containing LB agar plus 50 µg^−1^ of kanamycin, 1 mM IPTG, 5‐bromo‐4‐chloro‐3‐indolyl phosphate disodium salt (80 µg ml^−1^) and 6‐Chloro‐3‐indolyl‐β‐D‐galactopyranoside (100 µg ml^−1^). Cytoplasmic localization of the PhoA‐LacZ chimera is indicated by red/rose color development whereas translocation across the membrane is indicated by blue color development.

**Figure 5:**
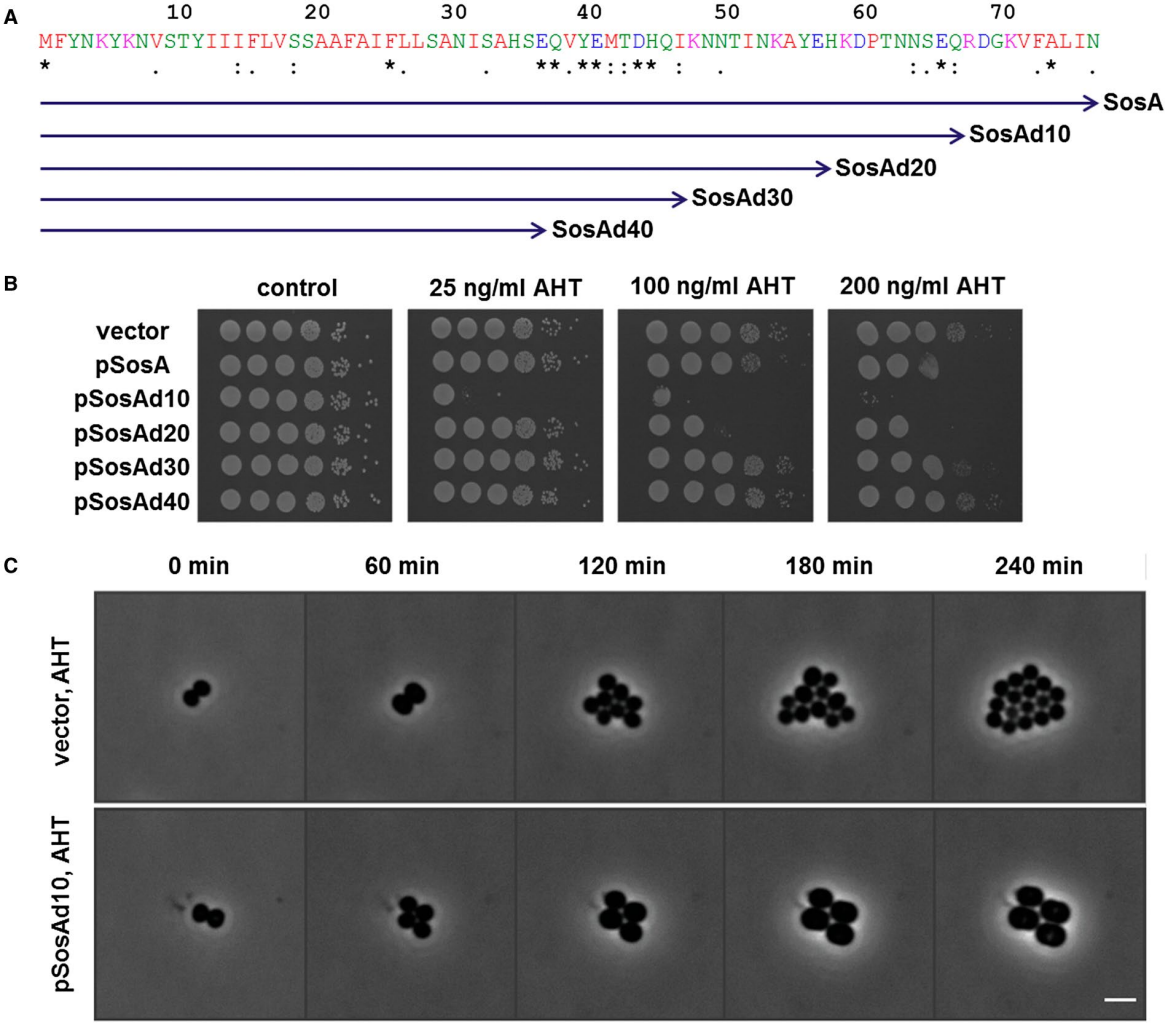
The effect of C‐terminal truncations of SosA on the inhibitory activity of the protein. A. Schematic representation of the different truncated SosA constructs. Full‐length SosA is a 77‐amino acid peptide. SosAd10 lacks the extreme C‐terminal 10 amino acids, while SosAd40 is SosA truncated of almost its entire extracellular C‐terminal part. Indicated conserved residues (*) originate from the alignment in Fig. 1. B. Activity of the constructs was assessed in *S. aureus* RN4220 and compared to the vector control (vector). Cells were grown exponentially to an OD_600_ of 0.5, serially 10‐fold diluted and plated on TSA (control) or TSA plus inducer (AHT) at indicated increasing concentrations followed by incubation overnight at 37°C. C. Visualization of the drastic cell size increase and the reduction in cell number of JE2/pRAB12‐*lacZ* (control) and JE2/pRAB12‐*sosAd10* in presence of 200 ng ml^−1^ of AHT by time‐lapse phase‐contrast microscopy at 37°C. Scale bar represents 2 µm.

**Figure 6:**
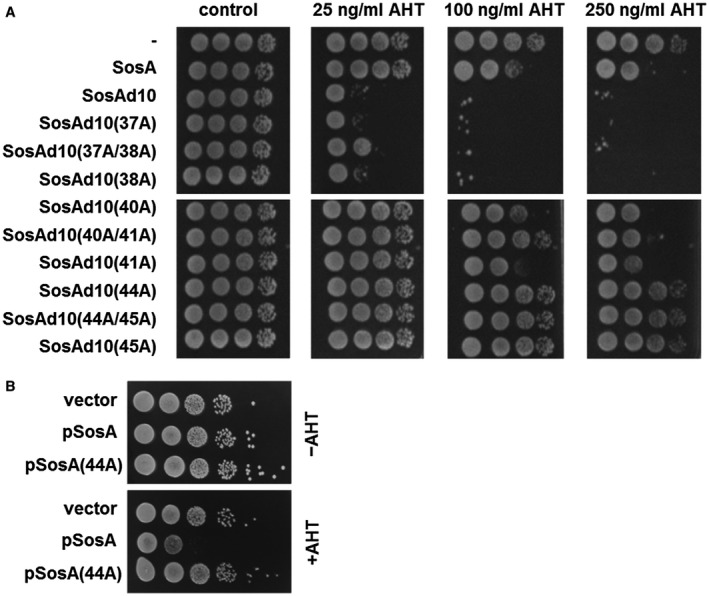
A. Assessment of the activity of SosAd10 variants with point mutations (alanine substitutions) at conserved residues (37/38, 40/41 and 44/45) within the C‐terminal part. Activity of the constructs was assessed in *S. aureus* JE2 and compared to the vector control (‐), SosA and SosAd10. Cells were grown exponentially to an OD_600_ of 0.5, serially 10‐fold diluted and plated on TSA (control) or TSA plus inducer (AHT) at increasing concentrations followed by incubation overnight at 37°C. B. Comparison of the activity of the point mutation protein SosA (44A) with WT SosA in *S. aureus* JE2. Cells were grown as above and plated on TSA or TSA plus 200 ng ml^−1^ AHT.

The C‐terminal part of SosA contains segments that appear somewhat conserved in staphylococci (Fig. 1B), and *in silico* it is predicted to be extracellular. First, we confirmed its extracellular location by constructing PhoA‐LacZ reporter fusions (Karimova and Ladant, 2017). Protein translocation to/across the membrane can be visualized using this system as LacZ remains enzymatically active only in the cytoplasm, whereas PhoA is active when in the periplasm. Here, a fusion of the reporter chimera to the C‐terminus of SosA conferred a phosphatase‐positive, β‐galatosidase‐negative phenotype in *E. coli*, indicative of translocation across the membrane (Fig. 4). Next, aiming at a functional characterization of its role in SosA activity, we constructed a series of C‐terminally truncated variants by the successive elimination of 10 AAs, resulting in four variants of SosA lacking between 10 and 40 of the C‐terminal AAs (Fig. 5A). Strikingly, we observed that truncation of the 10 C‐terminal AAs (and to some extent also removal of 20 C‐terminal AAs) strongly compromised plating efficiency even at low inducer concentrations (Fig. 5B). Time‐lapse microscopy revealed that cells producing SosAd10 (SosA missing the last 10 AAs) had a dramatic phenotype, with prominent cell swelling and a reduction of the cell cycle rate (Fig. 5C and Movie S3). The phenotypes from the plating assay correlated with the cell size measurements by flow cytometry (Fig. S2D). These results indicate that cells expressing the 10‐AA‐truncated proteins are stalled in a nondividing state leading to abnormal cell size, whereas those expressing full‐length SosA are only transiently halted or delayed in cell division (Fig. S2D and Movie S3). In contrast, deletion of 30 or 40 AAs from the C‐terminal part eliminated the ability of SosA to inhibit cell division, as cells expressing SosAd30 or SosAd40 gave comparable plating efficiency as those carrying the vector (Fig. 5B) and caused no effect on cell size upon induction (Fig. S2D). This prompted us to investigate the relevance of the highly conserved residues located in the membrane‐proximal part of the SosA C‐terminal part by performing alanine substitutions within the protein. To this end, we used the highly inhibitory SosAd10 variant as a scaffold and found that while point mutations at AA positions 37 or 38 (E and Q) had no effect, point mutations at positions 40 or 41 (Y and E) partially inactivated and the mutations at positions 44 and 45 (D and H) completely abolished the cell division inhibitory activity of the protein (Fig. 6A). The mutation at AA position 44 (D–A) was subsequently shown also inactivate the inhibitory activity of the full‐length protein (Fig. 6B). Importantly, the inactive variants SosAd40 and SosAd10 (44A) are still likely to be localized in the membrane, as are SosA and SosAd10, when evaluated by the PhoA‐LacZ translocation assay (Fig. 4). We conclude that conserved residues in the membrane‐proximal segment of the extracellular C‐terminal part of SosA are essential for inhibiting cell division, while truncation at the extreme C‐terminus of the protein augments activity.

### A CtpA protease mutant is hypersusceptible to SosA expression

**Figure 7:**
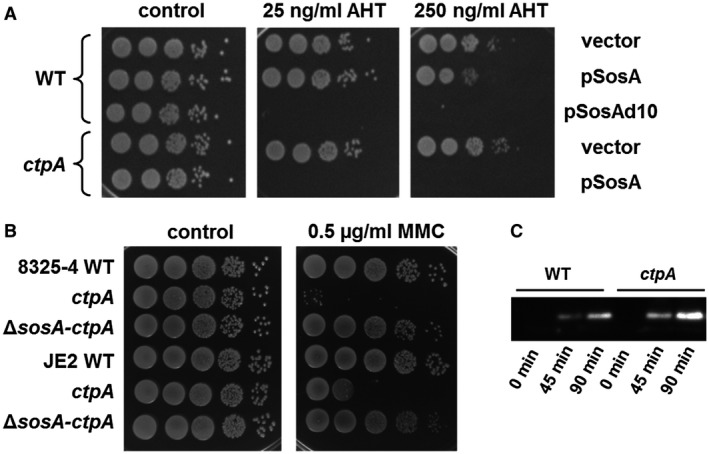
CtpA is a possible negative regulator of SosA. A. Hypersusceptibility of an *S. aureus* JE2 *ctpA* mutant to SosA‐mediated growth inhibition. Expression of *sosA* (pSosA) in WT *S. aureus* JE2 and the corresponding JE2‐*ctpA* mutant (*ctpA*) were compared and referenced to the vector control (vector) and expression of the hyperactive SosAd10 variant (pSosAd10) in the WT. Cells were grown exponentially to an OD_600_ of 0.5, serially 10‐fold diluted and plated on TSA (Control) or TSA plus inducer (AHT) at indicated concentrations. The plates were incubated overnight at 37°C and imaged. B. Assessment of mitomycin C susceptibility of a *ctpA* mutant by comparison of plating efficiency of *S. aureus* 8325‐4 and JE2 WT, *ctpA* and Δ*sosA*‐*ctpA* in presence of 0.5 µg ml^−1^ MMC. Cells were grown exponentially to an OD_600_ of 0.5 and serially 10‐fold diluted before plating and incubation at 37°C overnight. C. Western blot of accumulated SosA in *S. aureus* 8325‐4 WT and the *ctpA* mutant at indicated time points after the addition of 1 µg ml^−1^ of MMC to exponentially growing cells. The full blot is displayed in Fig. S5 and may serve as a control for equal loading and transfer by comparing intensities of nonspecific high molecular weight bands.

We reasoned that one explanation for the severe impact of the 10‐AA‐truncated SosA on cell division could be that the extreme C‐terminus of SosA serves as an endogenous signal for proteolytic degradation, and its removal allows the mutant protein to escape degradation and accumulate. Indeed, we observed that the cellular accumulation of the truncated protein was higher than for the WT protein following 1 h of ectopic expression (Fig. S2E). In parallel, we observed the same pattern for cells expressing the 10‐AA‐truncated SosA variant with the 44A mutation that abolishes the cell division inhibitory activity suggesting that the phenotype of the mutation is not related to instability of the SosA variant (Fig. S2E). To identify a membrane localized protease that may be responsible for SosA turnover, we searched the Nebraska Transposon Mutant Library (Fey *et al.*, 2013) for *S. aureus* protease mutant strains, and as a potential candidate, we identified the carboxyl‐terminal protease A, CtpA. The *S. aureus* CtpA protein is located at the cell membrane/cell wall fraction and is involved in stress tolerance and virulence in a mouse model of infection (Carroll *et al.*, 2014). Importantly, overproduction of the WT SosA protein in cells lacking *ctpA* completely abolished cell viability, similarly to what was observed when the 10‐AA‐truncated SosA protein was expressed in WT *S. aureus* cells (Fig. 7A). In agreement with this result, we found that overproduction of SosA and CtpA in the *ctpA* mutant background alleviated the detrimental effect of SosA and that the degree of complementation depended on the relative expressional levels of the two proteins, where higher expression level of SosA was only partially complemented by CtpA expression (Fig. S3A). Further supporting that the plating phenotype of the *ctpA* mutant is a direct consequence of cell division inhibition by SosA, we observed that the cell size distribution of the mutant upon SosA expression completely phenocopies the distribution obtained when expressing SosAd10 in the WT (Fig. S3B). Substantiating the negative regulatory role played by CtpA with respect to SosA, we noted that the *ctpA* mutant was substantially more sensitive to MMC (measured by plating efficiency) than WT *S. aureus*, but only when encoding a functional *sosA* gene (Fig. 7B). Additionally, upon MMC‐induced DNA damage, higher levels of the SosA protein accumulated in the *ctpA* mutant when compared to the WT (Fig. 7C). Importantly, parallel experiments were recently reported showing that CtpA in *B. subtilis* is one of two proteolytic regulators of the cell division inhibitor YneA, with CtpA displaying direct proteolytic activity against the target in vitro (Burby *et al.*, 2018). Altogether, based on these results, we propose that the extracellular C‐terminus of SosA is a substrate for CtpA proteolytic activity and that proteolysis relieves SosA‐mediated cell division inhibition allowing growth to resume once DNA stress has ceased.

### SosA is a late‐stage cell division inhibitor

**Figure 8:**
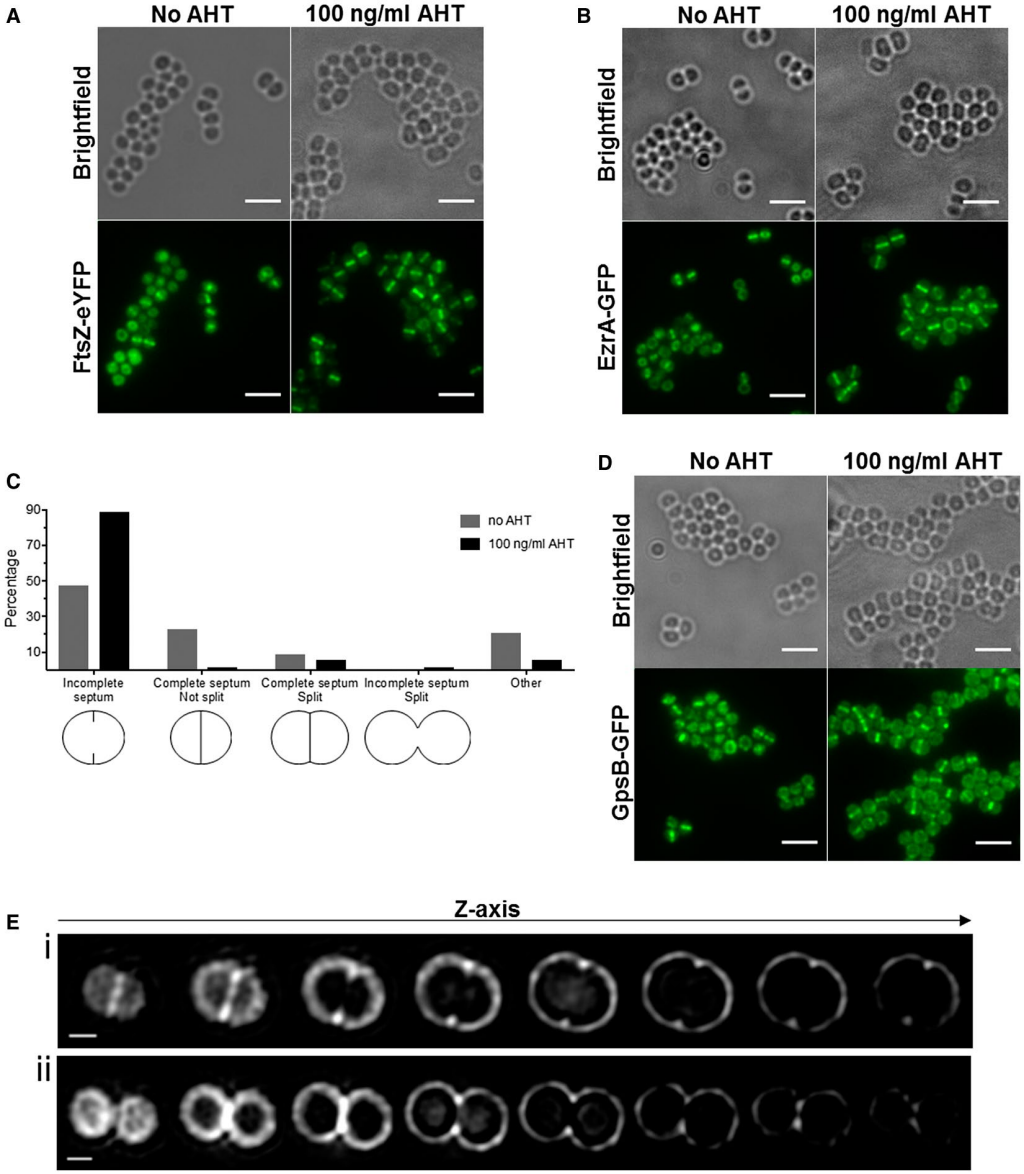
SosA does not impair the localization of cell division proteins and septum formation initiation. A. Localization of FtsZ‐eYFP in SJF4694 (JE2 pSosA pCQ11‐FtsZ‐eYFP) grown in the absence and presence of 100 ng ml^−1^ of AHT for 45 min. B. EzrA‐GFP localization in SJF4697 (JE2 pSosA *ezrA‐gfp*+) grown in the absence or presence of 100 ng ml^−1^ AHT for 45 min. C. Percentages of JE2 pSosA cells exhibiting incomplete, complete, complete split or incomplete split septa (n = 573 for no AHT, n = 607 for 100 ng ml^−1^ of AHT; organisms either isolated or co‐adhered in pairs; other represents staining in indistinct shape) after incubation with or without AHT for 45 min. D. GpsB‐GFP localization in SJF700 (JE2 pSosA *gpsB‐gfp*+) grown in the absence or presence of 100 ng ml^−1^ of AHT for 45 min. All fluorescence images (A, B and D) are average intensity projections and scale bars represent 3 um. E. 3D‐SIM Z‐stack images of JE2/pSosA grown with 100 ng ml^−1^ of AHT for 45 min and labeled with Alexa Fluor 647 NHS ester. (i) A cell with initiated septum formation and (ii) a cell splitting into daughter cells without finishing a septal disc. Scale bars represents 0.5 µm.

To pinpoint at which step of the cell division process the SosA‐mediated inhibition is taking place, we employed fluorescence microscopy to monitor the cellular localization of FtsZ and EzrA, crucial early stage cell division proteins in *S. aureus*, upon induction of SosA overproduction. Neither the *sosA* overexpression plasmid in noninducing conditions (Fig. 8A and B) nor the control vector in non‐inducing or inducing conditions (Fig. S4A and B) changed the cell morphology or protein localization. As expected, SosA overexpression caused cell swelling but also it did not cause de‐localization of the cell division initiator FtsZ (Fig. 8A) or affected the placement of the membrane‐bound division protein EzrA (Fig. 8B), which is an early recruited protein connecting the cytoplasmic division components with the peptidoglycan‐synthesizing membrane‐bound complex (Steele *et al.*, 2011). What was evident, however, was that whereas WT cells are heterogeneous with respect to FtsZ localization due to their presence in different phases of cell division, *sosA*‐expressing cells appear highly synchronized with FtsZ preferentially showing septal localization (compare uninduced and induced cells in Fig. 8A).

To further explore the cellular consequences of SosA‐mediated cell division inhibition, we examined the phenotypes of JE2/pSosA cells that were labeled for 5 min with HADA, marking regions of nascent peptidoglycan synthesis, and grown in the absence or presence of SosA induction (Fig. S4C). While in the absence of AHT, 47% and 23% of cells, respectively, showed a ring (incomplete septum) or line (complete septum) of nascent peptidoglycan synthesis, there was accumulation (89%) of cells with incomplete septa and a severe drop in cells with complete septa (1%) when SosA was overexpressed (Fig. 8C). Additionally, 1% of cells had an ‘hourglass’ shape after incubation with AHT, indicative of nonproductive, premature splitting taking place prior to septum completion. Next, whole cell walls of cells overproducing SosA were labeled with a fluorescent NHS ester and examined by 3D‐structured illumination microscopy (3D‐SIM). Microscopy visualization revealed that most cells had only signs of septation – a so‐called ‘piecrust’ (Turner *et al.*, 2010) (Fig. 8Ei) – and showed that in an hourglass‐like cell, there was a gap in the septal peptidoglycan (Fig. 8Eii). The fact that SosA does not affect localization of early cell division proteins, FtsZ and EzrA, and the cell population becomes synchronized to a particular stage of the cell cycle (phase P2 [Monteiro *et al.*, 2015]) shows that SosA does not hinder the initiation of septum formation but blocks the progression of septum completion – a phenotype similar to *S. aureus* DivIB‐depleted cells (Bottomley *et al.*, 2014). Although DivIB has been shown to be dispensable for FtsZ and EzrA localization to the septum and piecrust formation, its absence results in inhibition of septum progression and completion and in the mislocalization of GpsB (Bottomley *et al.*, 2014), a late cell division protein (Gamba *et al.*, 2009; Bottomley *et al.*, 2014). However, SosA overproduction did not impair the recruitment of GpsB to the midcell (Fig. 8D) even though visible cell enlargement occurred in this reporter strain (Fig. 8D) and not in the vector control (Fig. S4D). Altogether, these results indicate that SosA acts on (a) later cell division component(s) than FtsZ, EzrA and GpsB, and we conclude that SosA causes a characteristic halt in cell division by preventing progression through the morphological division stage P2, i.e. septum plate completion.

## Discussion

**Figure 9:**
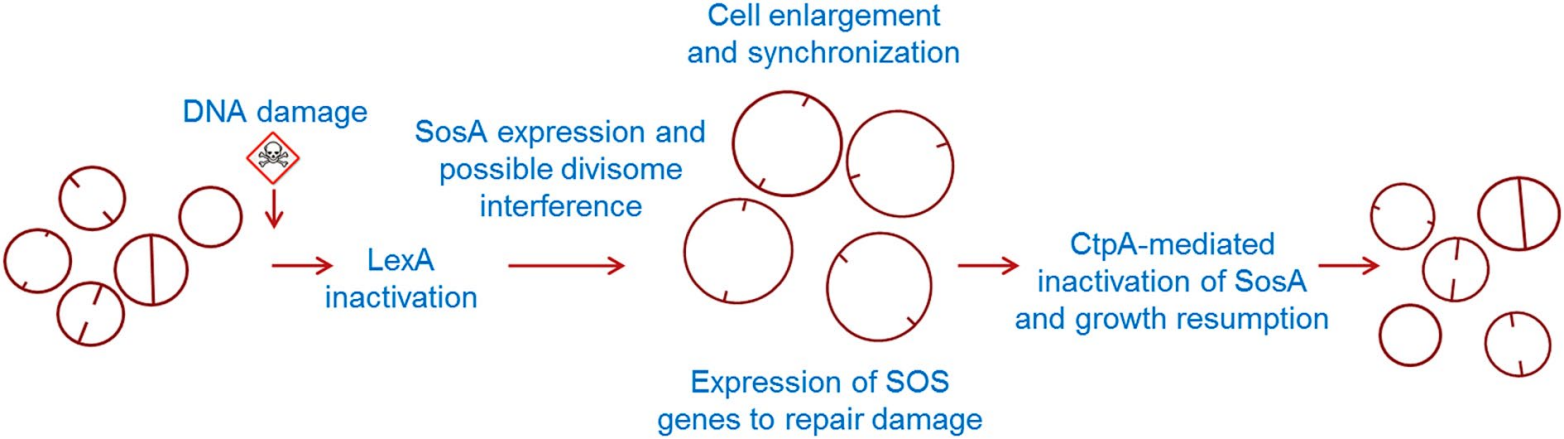
Proposed model for a regulated survival strategy for *S. aureus* upon DNA damage. As part of the SOS response, SosA is produced and, by a membrane‐localized activity, corrupts normal cell division activity, likely via an interaction with essential divisome components. In effect, cells are still able to initiate septum formation but are unable to complete it, leading to synchronization of the cell population. This provides a spatio‐temporal window for paralleled SOS response‐regulated DNA repair activity. At the same time, the cell size increases due to off‐septal activity of peptidoglycan‐synthesizing enzymes. As the SOS response diminishes, the cellular concentration of SosA is lowered, directly or indirectly, by the proteolytic activity of CtpA. At this stage, septa are allowed to complete and normal growth/division continues.

Based on the results reported here, we propose the following model for regulation of cell division in the prolate spheroid bacterium *S. aureus* under DNA‐damaging conditions (Fig. 9). During normal growth, the expression of *sosA* and the entire SOS regulon is repressed by the LexA repressor. Under DNA‐damaging and SOS‐inducing conditions, LexA is inactivated through autocleavage, which results in the expression of *sosA*. In this process, the N‐terminal autocleavage product of LexA needs to be further proteolytically processed for *sosA* expression to be fully induced (Cohn *et al.*, 2011), thus adding another layer of expressional control, suggesting tight regulation of the inhibitory protein. Upon derepression of LexA‐regulated genes, SosA accumulates to levels that inhibit cell division without causing FtsZ delocalization nor preventing the assembly of downstream components, EzrA and GpsB, which are representatives of early and later cell division components respectively. The inhibitor appears to affect the progression and completion of septum formation; consequently, the cells appear synchronized at the stage of septum formation initiation, while an ongoing off‐septal peptidoglycan synthesis leads to cell enlargement (Fig. S4C). Once conditions are favorable for continued division, *sosA* expression is repressed, the membrane protease CtpA degrades SosA and cell division resumes (Fig. 9).

The model illuminates several remaining unanswered questions. It remains to be shown at which point and how SosA affects divisome activity. Presently, no direct interaction has been observed between the Gram‐positive cell division inhibitors and FtsZ, the scaffold for cell division components assembly at the division site. Fluorescence microscopy suggests that also SosA does not act at this early stage (Fig. 8). Though poorly characterized mechanistically, the Gram‐positive inhibitors may suppress cell division at later stages, potentially by interacting with FtsI/FtsQ (for *M. tuberculosis* ChiZ) as suggested by bacterial two‐hybrid analysis (Vadrevu *et al.*, 2011) or via a pathway causing delocalization of FtsL and/or DivIC (for *B. subtilis* YneA) which was only suggested by indirect experimental data (Kawai and Ogasawara, 2006). These cell division inhibitors are characterized by the presence of a LysM peptidoglycan binding domain and for ChiZ also peptidoglycan hydrolase activity was suggested (Chauhan *et al.*, 2006). Such characteristics could provide important clues to their division inhibitory activity. Somewhat uniquely, however, a LysM domain appears to be absent from SosA. Another distinguishing feature of SosA is that FtsZ shows preferential septal localization upon expression (as a likely cause of synchronization), whereas for *B. subtilis* YneA, *M. tuberculosis* ChiZ and also *C. glutamicum* DivS, FtsZ ring formation was reported to be compromised upon expression of the respective cell division inhibitors (Kawai *et al.*, 2003; Chauhan *et al.*, 2006; Ogino *et al.*, 2008; Vadrevu *et al.*, 2011). Our preliminary characterization by two‐hybrid analysis suggests that SosA is capable of interacting with several essential *S. aureus* cell division components (data not shown), which is a subject for future experimental verification. Our microscopy and localization data suggest that SosA acts at a decision point between septum initiation and septum completion. In this regard, it is interesting that cell division in *S. aureus* was recently reported to be a two‐step process characterized by an initial, slow FtsZ‐dependent activity followed by a faster process when the lipid II flippase, MurJ, arrives and directs peptidoglycan synthesis to the septum (in division stage P2 where SosA expressing cells are stalled) (Monteiro *et al.*, 2018). More intriguingly, MurJ recruitment in *S. aureus* was reported to be mediated by the DivIB–FtsL–DivIC complex (Monteiro *et al.*, 2018), and this complex may serve as a central regulatory hub in bacterial cell division (den Blaauwen and Luirink, 2019). We hypothesize that this divisional stage could constitute an important molecular cue for the inhibitory activity of SosA, while we keep in mind that the inhibitory activity of SosA could be multifactorial and further studies are required to delineate the exact function of the protein.

Analysis of the amino acid sequence of SosA indicated that the N‐terminal part contains a TM domain, while the C‐terminal is located extracellularly. The hypothesis that the TM domain promotes localization to the cellular membrane was supported by PhoA‐LacZ fusion to SosA and by various point and deletion mutants of the fusion protein (Fig. 4). The abrogation of activity by the 40‐AA truncation as well as by a single alanine substitution (D44A) suggests that this segment is essential for correct localization to the septum or, perhaps more interestingly, that the actual motif for cell division inhibition is localized at the exterior of the membrane. It should be noted that the PhoA–LacZ assay monitors protein translocation in *E. coli* and at present we cannot exclude that the (D44A) point mutation affects membrane translocation or stability within the membrane in *S. aureus*. Future work should reveal the extent to which this highly conserved residue is mechanistically important for SosA activity. If divisome interference indeed lies within this extracellular segment of SosA, it could provide a novel paradigm for interference with cell division beyond the transpeptidase‐inhibitory activity exerted by β‐lactam antibiotics.

Endogenously triggered cell division inhibition must be strictly regulated, as demonstrated by pioneering work showing that SulA of *E. coli* is degraded by the Lon protease (Mizusawa and Gottesman, 1983). Similarly, YneA from *B. subtilis* was originally reported to be regulated by proteolysis, although by an undefined mechanism, and a point mutation at its extreme C‐terminus generated a stabilized yet functional variant of the cell division inhibitor (Mo and Burkholder, 2010). In *B. megaterium*, the temporal expression of a YneA homologue has been suggested to be due to mRNA instability (Buchholz *et al.*, 2013). We demonstrate that a minor truncation of the C‐terminus of SosA increases cell division inhibitory activity (Fig. 5), possibly as a consequence of accumulation of the protein (Fig. S2E). This led us to speculate that the extreme C‐terminus serves as a signal for proteolysis and we identified the CtpA protease‐deficient strain in which SosA appeared to accumulate and had an increased detrimental effect compared to the WT background. Bacterial carboxy‐terminal proteases are poorly characterized, particularly with respect to their substrate preferences; however, we propose that CtpA specifically regulates SOS‐induced cell division inhibition in staphylococci, likely via recognition of the C‐terminus of SosA. In support of this, Burby *et al.* (2018) recently disclosed that CtpA is involved in turnover of YneA in *B. subtilis*. Of note, carboxy‐terminal proteases are ubiquitous in bacterial genera, and homologs of CtpA are found in other Gram‐positive species, including also *L. monocytogenes* which share a homolog of YneA. Hence, the CtpA‐like proteases could represent functional homologs of the *E. coli* Lon protease, allowing negative regulation of membrane‐localized endogenous cell division inhibition more broadly among Firmicutes and beyond.

SosA may also prove to be a useful tool for scientists conducting studies of bacterial cell division, which have recently benefitted from the development of genetic tools and highly sophisticated microscopy imaging technologies. Most often bacteria grow as an unsynchronized population with individual cells in different phases of cell division, making it challenging to order the temporal events that occur during cell cycle progression, DNA replication and division. SosA expression is halting the *S. aureus* cell cycle at a specific morphological stage, e.g. following the piecrust formation, and we anticipate that the exploitation of this function may constitute a useful tool for the generation of synchronized staphylococcal cells for experimental purposes.

Apart from its basic biological interest, the bacterial cell division process may constitute an unexhausted source of novel therapeutic targets (Lock and Harry, 2008; Sass and Brötz‐Oesterhelt, 2013). We believe that SosA could be a natural scaffold, and that further studies on this protein can provide new therapeutically accessible targets that would interfere with an essential process in *S. aureus* and other staphylococci.

## Experimental procedures

### Bacterial strains and plasmids used in this work

The bacterial strains and plasmids used in this study are listed in Table S1. *E. coli* strains were grown in Luria–Bertani medium (Oxoid) or in LB agar (Oxoid). *S. aureus* strains were grown in Tryptic Soy Broth (TSB) or in Tryptic Soy Agar (TSA) (both Oxoid). Ampicillin (100 µg ml^−1^), chloramphenicol (10 µg ml^−1^) or erythromycin (5 µg ml^−1^) were added when appropriate. Anhydrotetracycline (AHT) (Sigma‐Aldrich) and Isopropyl β‐D‐1‐thiogalactopyranoside (IPTG) (Thermo Scientific) were used as inducers for protein expression.

### Construction of strains and plasmids

All oligonucleotides used are listed in Table S1. *S. aureus* 8325‐4Δ*sosA* is a clean deletion of *sosA* (SAOUHSC_01334) in strain 8325‐4 obtained by allelic replacement using the temperature‐sensitive shuttle vector pIMAY (Monk *et al.*, 2012). The 1 kb regions up‐ and downstream of *sosA* were PCR amplified (Phusion Hot Start II DNA Polymerase, Thermo Scientific) using primer pairs Up‐sosA_fw‐KpnI/Up‐sosA_rev and Dw‐sosA_fw/Dw‐sosA_rev‐SacI, respectively, and subsequently joined in a spliced overhang PCR using Up‐sosA_fw‐KpnI/Dw‐sosA_rev‐SacI. The resulting deletion fragment was cloned into pIMAY via KpnI/SacI, generating pIMAY‐Δ*sosA*, purified from *E. coli* DC10B and transformed into *S. aureus* subsequently maintained at 28°C. Chromosomal integration of the plasmid was performed at 37°C under selective conditions (chloramphenicol) followed by passage at 28°C without antibiotic selection and final plating on TSA containing 500 ng ml^−1^ of AHT for plasmid counterselection. Colonies were replica‐plated to select for sensitivity toward chloramphenicol and successful allelic exchange were screened for by PCR amplification using primer pairs Ctrl_dsosA_F/Ctrl_dsosA_R positioned outside *sosA* and Fwd_MCS/Rev_MCS targeting the vector respectively. The allelic replacement procedure was identical for *S. aureus* JE2 to create JE2Δ*sosA*.

The chromosomal transposon insertion mutation in *ctpA* conferring erythromycin resistance was obtained from the Nebraska Transposon Mutant Library (NTML, NE847) (Fey *et al.*, 2013) and was moved by transduction (phage Φ11) to *S. aureus* JE2, JE2Δ*sosA*, S*. aureus* 8325‐4 and 8325‐4Δ*sosA*, resulting in JE2‐*ctpA*, JE2Δ*sosA*‐*ctpA*, 8325‐4‐*ctpA* and 8325‐4Δ*sosA*‐*ctpA* respectively. JE2‐*ctpA*(−erm), an erythromycin‐sensitive derivative, was generated by elimination of the transposon‐encoded *ermB* in JE2‐*ctpA* by allelic exchange using the pTnT vector using temperature‐mediated chromosomal integration and *secY*‐mediated counterselection as described before (Bose *et al.*, 2013).

All plasmid constructs mentioned below were cloned in *E. coli* IM08B (Monk *et al.*, 2015) from where they were transformed into *S. aureus* strains. For complementation of the *sosA* knockout, the *sosA* gene with its native promoter was PCR amplified from strain 8325‐4 using primers Up‐sosA‐promo_SalI and Dw‐sosA_EcoRI and cloned into designated restriction sites in plasmid pJC1112 (Chen *et al.*, 2014). The resulting plasmid (pJC1112‐*sosA*) was transformed into the integration‐proficient strain RN9011, creating RN9011‐*sosA*‐compl. when selected with erythromycin. The chromosomally integrated plasmid was transduced into 8325‐4Δ*sosA*, resulting in 8325‐4Δ*sosA*‐compl. For *sosA* expression the *sosA* gene including its predicted ribosomal binding site was cloned into the BglII and EcoRI sites of pRAB12‐*lacZ* (Helle *et al.*, 2011) using primers Up‐sosA_BglII/Dw‐sosA_EcoRI, at the same time eliminating the *lacZ* reporter gene. The resulting plasmid was called pSosA. Generation of plasmids encoding C‐terminally truncated variants of SosA was obtained by PCR amplification of DNA fragments using Up‐sosA_BglII and downstream primers Dw‐sosA(d10)_EcoRI to Dw‐sosA(d40)_EcoRI, all equipped with premature stop codons. The PCR products were ligated into pRAB12‐*lacZ* using BglII and EcoRI cut sites to create pSosAd10, pSosAd20, pSosAd30 and pSosAd40. Single and double amino acid substitutions (alanine) in the SosAd10 protein were obtained by cloning commercially synthesized DNA fragments (Twist Bioscience) into pRAB12‐*lacZ* using the same restriction sites. The mutation at AA position 44 (D to A) was introduced to the full length SosA protein via extension PCR by first using primers Up‐sosA_BglII/SosA_R‐long on the SosAd10(44A) template, then using that product as template for amplification with primers Up‐sosA_BglII/Dw‐sosA_EcoRI followed by cloning into pRAB12‐*lacZ*, thereby generating pSosA(44A). Plasmid pCtpA was constructed by cloning the *ctpA* gene behind the P_spac_ promoter in pSK9067 (Brzoska and Firth, 2013) using primer pair ctpA_F‐SalI/ctpA_R‐EcoRI. For membrane topology analysis using pKTop (Karimova and Ladant, 2017), *sosA*, *sosAd10*, *sosAd10(44A)* and *sosAd40* were PCR amplified from respective expression constructs using primer SosA_F‐BamHI and primers SosA_R‐KpnI, SosAd10_R‐KpnI or SosAd40_R‐KpnI, respectively, and cloned in frame in front of the *phoA‐lacZ* chimeric gene using BamHI/KpnI restriction sites.

In order to construct JE2 strains producing a fluorescent fusion of FtsZ, EzrA or GpsB in the presence of the *sosA* expression plasmid, JE2 pSosA was transduced with a lysate from SH4665 (SH1000 pCQ11‐FtsZ‐eYFP), JGL227 (SH1000 *ezrA‐gfp*+) or JGL228 (SH1000 *gpsB‐gfp*+), resulting in SJF4694 (JE2 pRAB12‐*lacZ* pCQ11‐FtsZ‐eYFP), SJF4696 (JE2 pRAB12‐*lacZ ezrA‐gfp*+) and SJF4699 (JE2 pRAB12‐*lacZ gpsB ‐gfp*+) respectively. Control strains SJF4693 (JE2 pRAB12‐*lacZ* pCQ11‐FtsZ‐eYFP), SJF4696 (JE2 pRAB12‐*lacZ ezrA‐gfp*+) and SJF4699 (JE2 pRAB12‐*lacZ gpsB‐gfp*+) were constructed by a phage transduction of JE2 pRAB12‐*lacZ* with lysates from SH4665 (SH1000 pCQ11‐FtsZ‐eYFP), JGL227 (SH1000 *ezrA‐gfp*+) or JGL228 (SH1000 *gpsB‐gfp*+) respectively. To induce FtsZ‐eYFP production in SJF4693 and SJF4694, cells were grown in the presence of 50 μM IPTG.

### Determination of OD_600_ and CFU after MMC treatment

Strains were grown overnight on TSA plates and used for inoculating TSB and allowed to grow to OD_600_ = 0.05 when Mitomycin C (MMC from *Streptomyces caespitosus*, Sigma‐Aldrich) was added. Cell density was monitored by OD_600_ measurements at intervals onward and samples were withdrawn to determine culture colony forming units by serial dilution in 0.9% w/v NaCl and plating on TSA.

### Flow cytometry

Cell size distributions of cultures were arbitrarily quantified by flow cytometry using the forward scatter signal (FSC‐A) acquired on a BD Accuri™ C6 flow cytometer (BD Biosciences). Cell samples were diluted in 0.9% w/v NaCl to an approximate density of 10^6^ cells ml^−1^ and sonicated briefly to allow acquisition of scatter signal from single cells predominantly. Sonication was performed with a Bandelin sonopuls HD2070/UW2070 apparatus (Bandelin electronics, Germany) fitted with the MS 73 probe. Ten pulses of 500 ms were given at 50% power. All flow cytometry experiments were independently repeated at least twice with similar results.

### Plating assays

Spot dilution was used to evaluate plating efficiency of strains carrying various plasmid constructs. Strains were grown exponentially until an approximate OD_600_ of 0.5 under plasmid‐selective conditions. Strains were then 10‐fold serially diluted in 0.9% w/v NaCl and positioned as 10 µl spots on TSA containing selective antibiotics with/without inducer at indicated concentrations and incubated at 37°C overnight. All spot plating assays were independently repeated with similar results.

### Labeling *S. aureus* with HADA

Cells grown to mid‐exponential phase (OD_600_ ~ 0.5) were incubated with 500 µM HADA at 37°C for 5 min. Cells were then washed by centrifugation and resuspension in PBS.

### Labeling *S. aureus* with NHS ester

Labeling with NHS ester was performed as described before (Lund *et al.*, 2018). Briefly, cells grown to mid‐exponential phase (OD_600_ ~ 0.5) were collected by centrifugation and growth medium was discarded. Cells were resuspended in PBS containing 8 μg ml^−1^ Alexa Fluor 647 NHS ester (Invitrogen) and incubated at room temperature for 5 min. Cells were washed by centrifugation and resuspension in PBS.

### Fixing

Cells were fixed by incubation in 4% (w/v) paraformaldehyde at room temperature for 30 min.

### Widefield epifluorescence microscopy

Fixed cells were dried onto a poly‐L‐Lysine coated slide and mounted in PBS. Imaging was performed using either a Nikon Ti Inverted microscope fitted with a Lumencor Spectra X light engine or a v4 DeltaVision OMX 3D‐SIM system (Applied Precision, GE Healthcare, Issaquah, USA).

### 3D‐structured illumination microscopy

A high‐precision cover slip (High‐precision, No. 1.5 H, 22 × 22 mm, 170 ± 5 µm, Marienfeld) was cleaned by sonicating in 1 M KOH for 15 min at room temperature. The coverslip was washed with water and incubated in 0.01% (w/v) poly‐L‐lysine solution (Sigma) for 30 min at room temperature. The coverslip was rinsed with water and dried with nitrogen. Fixed cells were dried onto the poly‐L‐Lysine coated cover slip and mounted on a slide with SlowFade Diamond (Invitrogen). 3D SIM visualization was performed using a v4 DeltaVision OMX 3D‐SIM system (Applied Precision, GE Healthcare, Issaquah, USA) equipped with a Plan Apo 60x, 1.42 NA oil objective, using 1.514 immersion oil, a 642 nm laser and a standard excitation/emission filter set (683/40). For each z‐section, a sample was imaged in five phase shifts and three angles. The z‐sections were 0.125 nm in depth. Raw data were reconstructed with the Softworx software (GE Healthcare) using OTFs optimized for the specific wavelength and oil used.

### Time‐lapse microscopy

Strains were grown overnight in TSB medium at 37°C, then diluted 100 times in fresh TSB and grown until OD = 0.1. Cells were washed once with fresh TSB and spotted onto TSB–acrylamide (10%) pads previously incubated for 2 h in TSB medium supplemented, when appropriate, with 0.04 µg ml^−1^ mitomycin C (MMC) or 200 ng ml^−1^ anhydrotetracycline (AHT). Pads were placed into a Gene frame (Thermo Fisher Scientific) and sealed with a cover glass. Phase‐contrast images were acquired on a DV Elite microscope (GE healthcare) equipped with a sCMOS (PCO) camera and a 100x oil immersion objective. Images were acquired with 200 ms exposure time every 4 min for at least 6 h at 37°C using Softworx (Applied Precision) software. Images were analyzed using Fiji (http://fiji.sc).

### Western blot analysis

Purified rabbit anti‐SosA antibody was obtained via GenScript Biotech (Netherlands). A custom peptide, CEHKDPTNNSEQRDGA, comprising AAs 57–70 of SosA was used for immunization. Bacterial cells were pelleted and frozen immediately at −80°C before being resuspended in PBS containing cOmplete™ Mini Protease Inhibitor Cocktail (Sigma‐Aldrich) and lysed by bead beating (Fastprep‐24™, MP Biomedicals). The protein concentration of the lysates was measured using the Qubit™ Protein Assay Kit (ThermoFisher Scientific) and samples were normalized to equal amounts of protein, 20 µg of total protein was separated on NuPAGE^®^ 4‐12% Bis‐Tris gels using MES buffer and the XCell sure‐lock mini‐cell system (ThermoFisher Scientific), transferred to a polyvinylidene difluoride membrane (PVDF) and probed with primary antibody diluted 1:1000. Bound antibody was detected with the WesternBreeze^®^ Chemiluminescent Kit, anti‐rabbit according to the instructions from the manufacturer (ThermoFisher Scientific). All western blots were independently repeated with similar results. Uncropped versions of blots are displayed in Fig S5.

## Author contributions

M.S.B., K.W., P.K., M.T.C., S.J.F. and H.I. conceived and designed the study. Experiments were performed by M.S.B., K.W., C.G. and A.L.B. M.S.B. K.W., C.G., G.L., D.F., J.‐W.V., S.J.F. and H.I. contributed to analysis of data and drafting of the manuscript. All authors read and approved the final manuscript.
