# Supplementary material for: SosA inhibits cell division in Staphylococcus aureus in response to DNA damage
Source: Mol Microbiol. 2019 Aug 16;112(4):1116–30. doi: 10.1111/mmi.14350 (PMC6851548; doi:10.1111/mmi.14350)
Supplement: Supplementary file 1 [file MMI-112-1116-s001.docx]

**Supplemental Material for**

**SosA inhibits cell division in *Staphylococcus aureus* in response to DNA damage**

**Martin S. Bojer, Katarzyna Wacnik, Peter Kjelgaard, Clement Gallay, Amy L. Bottomley, Marianne T. Cohn, Gunnar Lindahl, Dorte Frees, Jan-Willem Veening, Simon J. Foster & Hanne Ingmer**

**Supplementary Table S1.**

1. Bacterial strains used (*S. aureus* if not otherwise indicated).

| **Name** | **Characteristics** | **Source/**  **Reference** |
| --- | --- | --- |
| *E. coli* DC10B | Lab strain used for cloning | [1] |
| *E. coli* IM08B | Lab strain used for cloning | [2] |
| RN4220 | Lab strain, resitriction modification-deficient | Lab stock |
| 8325-4 | Lab strain | Lab stock |
| 8325-4Δ*sosA* | Clean deletion of *sosA* in 8325-4 | This study |
| 8325-4Δ*sosA*-compl. | Complementation of 8325-4Δ*sosA* at *attC* site; Erm^R^ | This study |
| 8325-4-*ctpA* | Transposon insertion in *ctpA* of 8325-4 transduced from NE847 [3]; Erm^R^ | This study |
| 8325-4Δ*sosA*-*ctpA* | Transposon insertion in *ctpA* of 8325-4 Δ*sosA* transduced from NE847 [3]; Erm^R^ | This study |
| JE2 | Lab strain | Lab stock |
| JE2Δ*sosA* | Clean deletion of *sosA* in JE2 | This study |
| JE2-*ctpA* | Transposon insertion in *ctpA* of JE2, transduced from NE847 [3]; Erm^R^ | This study |
| JE2-*ctpA*(−erm) | Erm-sensitive derivative of JE2-*ctpA* | This study |
| JE2Δ*sosA*-*ctpA* | Transposon insertion in *ctpA* of JE2 Δ*sosA*, transduced from NE847 [3]; Erm^R^ | This study |
| RN9011 | RN4220/pRN7023 (SaPI1 integrase, cat194) | [4] |
| RN9011-*sosA*-compl. | Integration of pJC1112-*sosA* at *attC* site; Erm^R^ | This study |
| SH4665 | SH1000 pCQ11-FtsZ-eYFP; Erm^R^ | [5] |
| JGL227 | SH1000 *ezrA-gfp*+; Erm^R^ | [6] |
| JGL228 | SH1000 *gpsB -gfp*+; Erm^R^ | [6] |
| SJF4693 | JE2 pRAB12-*lacZ* pCQ11-FtsZ-eYFP; Cm^R^, Erm^R^ | This study |
| SJF4694 | JE2 pSosA pCQ11-FtsZ-eYFP; Cm^R^, Erm^R^ | This study |
| SJF4696 | JE2 pRAB12-*lacZ* *ezrA-gfp*+; Cm^R^, Erm^R^ | This study |
| SJF4697 | JE2 pSosA *ezrA-gfp*+; Cm^R^, Erm^R^ | This study |
| SJF4699 | JE2 pRAB12-*lacZ* *gpsB-gfp*+; Cm^R^, Erm^R^ | This study |
| SJF4700 | JE2 pSosA *gpsB-gfp*+; Cm^R^, Erm^R^ | This study |

1. Plasmids used.

| **Name** | **Characteristics** | **Source/**  **Reference** |
| --- | --- | --- |
| pRAB12-*lacZ* | Expression vector; Cm^R^ | [7] |
| pIMAY | Vector for temperature-sensitive allelic replacement; Cm^R^ | [1] |
| pIMAY-*ΔsosA* | *ΔsosA* deletion fragment cloned into pIMAY; Cm^R^ | This study |
| pSosA | *sosA* cloned behind anhydrotetracycline-inducible promoter of pRAB12-*lacZ;* Cm^R^ | This study |
| pSosAd10 | *sosAd10* cloned behind anhydrotetracycline-inducible promoter of pRAB12-*lacZ;* Cm^R^ | This study |
| pSosAd20 | *sosAd20* cloned behind anhydrotetracycline-inducible promoter of pRAB12-*lacZ;* Cm^R^ | This study |
| pSosAd30 | *sosAd30* cloned behind anhydrotetracycline-inducible promoter of pRAB12-*lacZ;* Cm^R^ | This study |
| pSosAd40 | *sosAd40* cloned behind anhydrotetracycline-inducible promoter of pRAB12-*lacZ;* Cm^R^ | This study |
| pSK9067 | Expression vector; Erm^R^ | [8] |
| pCtpA | *ctpA* cloned into pSK9067, IPTG-inducible; Erm^R^ | This study |
| pJC1112 | Vector for integration into *attC* site; Erm^R^ | [4] |
| pJC1112-*sosA* | *sosA* behind its own promoter cloned into pJC1112; Erm^R^ | This study |
| pTnT | Vector for elimination of Erm^R^ from transposon insertion | [9] |
| pSosAd10(37A) | Alanine substitution variant of SosAd10 cloned into pRAB12-*lacZ*; Cm^R^ | This study |
| pSosAd10(37A/38A) | Alanine substitution variant of SosAd10 cloned into pRAB12-*lacZ*; Cm^R^ | This study |
| pSosAd10(38A) | Alanine substitution variant of SosAd10 cloned into pRAB12-*lacZ*; Cm^R^ | This study |
| pSosAd10(40A) | Alanine substitution variant of SosAd10 cloned into pRAB12-*lacZ*; Cm^R^ | This study |
| pSosAd10(40A/41A) | Alanine substitution variant of SosAd10 cloned into pRAB12-*lacZ*; Cm^R^ | This study |
| pSosAd10(41A) | Alanine substitution variant of SosAd10 cloned into pRAB12-*lacZ*; Cm^R^ | This study |
| pSosAd10(44A) | Alanine substitution variant of SosAd10 cloned into pRAB12-*lacZ*; Cm^R^ | This study |
| pSosAd10(44A/45A) | Alanine substitution variant of SosAd10 cloned into pRAB12-*lacZ*; Cm^R^ | This study |
| pSosAd10(45A) | Alanine substitution variant of SosAd10 cloned into pRAB12-*lacZ*; Cm^R^ | This study |
| pSosA(44A) | Alanine substitution variant of full length SosA in pRAB12-*lacZ*; Cm^R^ | This study |
| pKTop | Vector with *phoA-lacZ* fusion for membrane topology analysis; Kan^R^ | [10] |
| pKTop-s*osA* | *sosA* cloned in frame into pKTop; Kan^R^ | This study |
| pKTop-*sosAd10* | *sosAd10* cloned in frame into pKTop; Kan^R^ | This study |
| pKTop-*sosAd10(44A)* | *sosAd10(44A)* cloned in frame into pKTop; Kan^R^ | This study |
| pKTop-*sosAd40* | *sosAd40* cloned in frame into pKTop; Kan^R^ | This study |

1. Oligonucleotides used.

| **Name** | **Sequence (5’-3’)** |
| --- | --- |
| Up-sosA_fw-KpnI | ATATGGTACCCTCGCTCCTGTAAATTATTACG |
| Up-sosA_rev | TTTCACTCCTAGAACATTTGTTTG |
| Dw-sosA_fw | CAAATGTTCTAGGAGTGAAATACATTGTCACAACGTTATTTTG |
| Dw-sosA_rev-SacI | ATATGAGCTCCATATGTGTAATGATCTACAACATTATATC |
| Ctrl_dsosA_F | ATTCTCTCATATATAGGCACTCC |
| Ctrl_dsosA_R | CTGTTTGCTCCTTTGCTTC |
| Fwd_MCS | TACATGTCAAGAATAAACTGCCAAAGC |
| Rev_MCS | AATACCTGTGACGGAAGATCACTTCG |
| ctpA_F-SalI | ATATGTCGACCATAATAAGGAAGTGATACAATGG |
| ctpA_R-EcoRI | GATACAGAATTCTACAATTTTAGTAGTGTGTATCGC |
| Up-sosA-promo_SalI | GATACAGTCGACCTCTCATATATAGGCACTCCC |
| Up-sosA_BglII | GATACAAGATCTGTTCTAGGAGTGAAAATGATG |
| Dw-sosA_EcoRI | GATACAGAATTCTCAATTTATTAAAGCGAACAC |
| Dw-sosA(d10)_EcoRI | GATACAGAATTCTCATTGTTCGCTATTGTTTGTAG |
| Dw-sosA(d20)_EcoRI | GATACAGAATTCTCATTCGTATGCTTTATTTATCGT |
| Dw-sosA(d30)_EcoRI | GATACAGAATTCTCAAATTTGATGGTCAGTCATTTC |
| Dw-sosA(d40)_EcoRI | GATACAGAATTCTCATTCCGAGTGAGCACTAATG |
| SosA_R-long | ATTTATTAAAGCGAACACTTTCCCATCTCTTTGTTCGCTATTGTTTGTAG |
| SosA_F-BamHI | GATACAGGATCCCATGTTTTACAATAAATATAAAAACG |
| SosA_R-KpnI | GATACAGGTACCTCATTTATTAAAGCGAACACTTT |
| SosAd10_R-KpnI | GATACAGGTACCTCTTGTTCGCTATTGTTTGTAG |
| SosAd40_R-KpnI | GATACAGGTACCTCTTCCGAGTGAGCACTAATG |

**Supplementary Figure S1.**


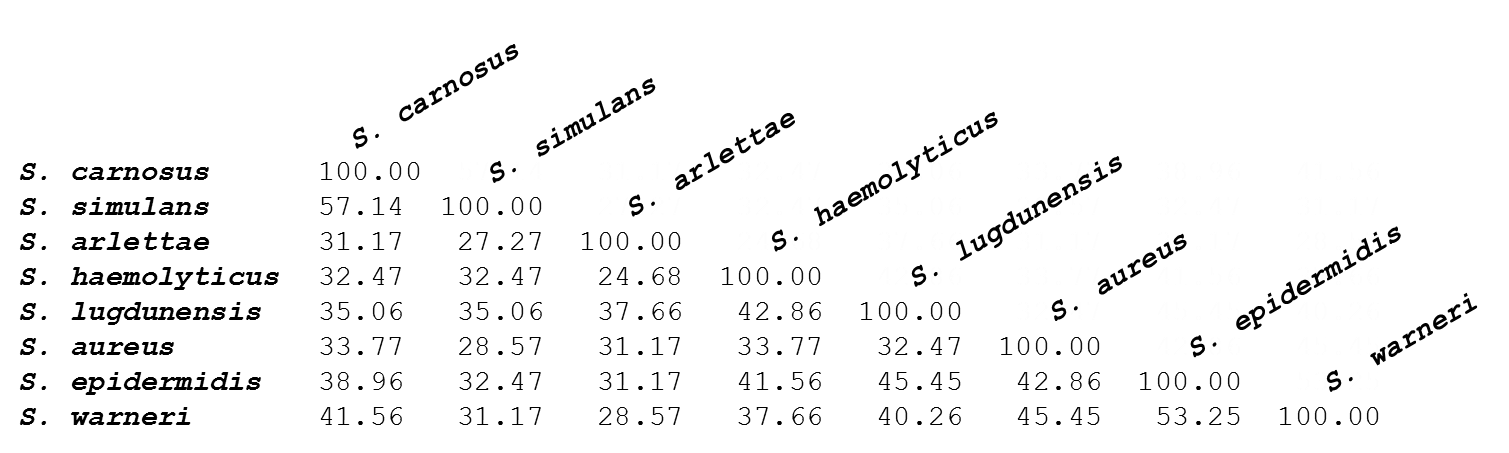


**Fig. S1** Percent identity matrix for staphylococcal SosA proteins. Pairwise identity scores for the proteins included in the alignment in Figure 1 were obtained by the Clustal 2.1 algorithm.

**Supplementary Figure S2.
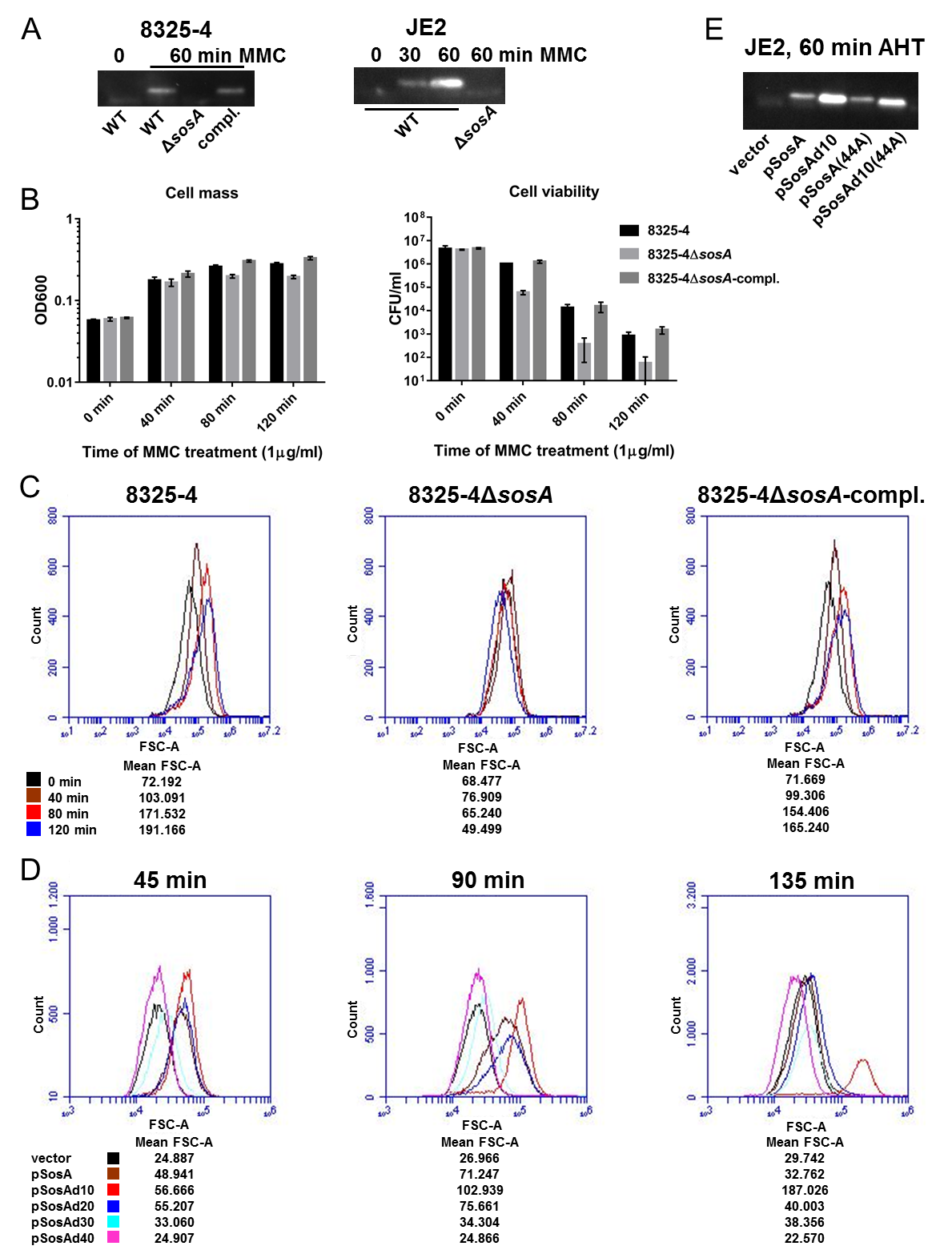
**

**Fig. S2** (A) Detection by western blot of DNA-damage induced expression of SosA. SosA being detected in wildtype *S. aureus* 8325-4 and the complemented strain, while being absent in the *sosA* deletion mutant following 1 h of MMC treatment (1 µg/ml). Also displayed is the time-dependent expression of SosA in *S. aureus* JE2 post MMC addition (1 µg/ml) in comparison to the corresponding *sosA* deletion mutant. (B and C) Phenotypic complementation of the *sosA* deletion mutant. The phenotypes of *S. aureus* 8325-4Δ*sosA* were restored back to wildtype by chromosomal integration of a copy of the *sosA* gene under its native promoter (*S. aureus* 8325-4Δ*sosA*-compl.) when evaluated for changes in cell density and viability (B) and cell size assessed by flow cytometry (C) during challenge with MMC (1 µg/ml). Mean FSC-A values are indicated below histograms. Cells were grown exponentially prior to addition of MMC at an OD_600_ of 0.05. (D) Effect of different truncated SosA variants on *S. aureus* cell size. Evaluation of cell size distribution was performed by flow cytometry (FSC-A) of *S. aureus* RN4220 containing expression plasmids encoding full length SosA or C-terminally truncated variants of the protein. Mean FSC-A values are indicated below histograms. Cells were grown exponentially prior to induction with 100 ng/ml of AHT and analyzed at indicated time points. (E) Western blot of accumulation in *S. aureus* JE2 of SosA, SosAd10, SosA(44A), and SosAd10(44A) expressed from respective plasmid constructs for 1 h with 200 ng/ml AHT. The full blots for A and E are displayed in Fig. S5 and may serve as a control for equal loading and transfer by comparing intensities of non-specific high molecular weight bands.

**Supplementary Figure S3.**

**
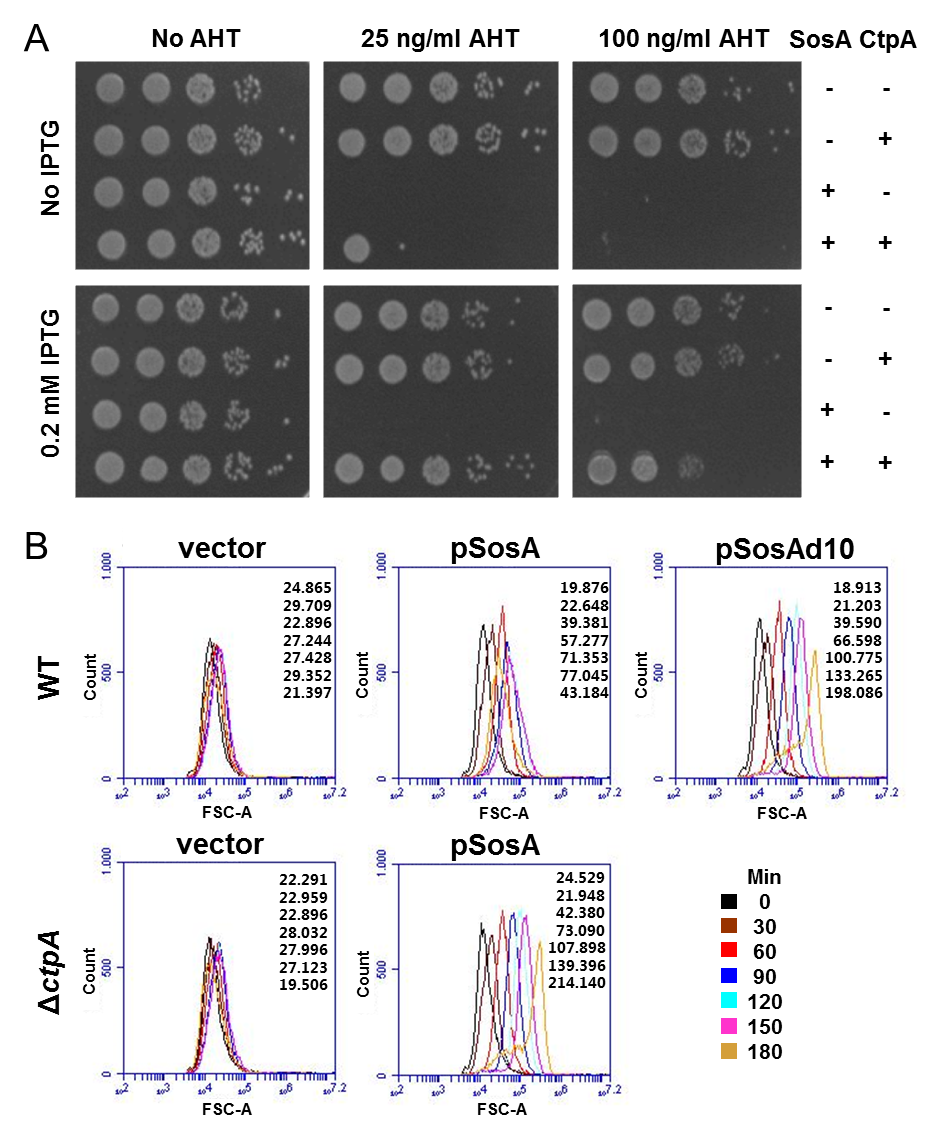
**

**Fig. S3** Hypersusceptibility of an *S. aureus* JE2 *ctpA* mutant to SosA-mediated growth inhibition. (A) Plating efficiency of the *S. aureus* JE2-*ctpA*(−erm) mutant transformed with both SosA and CtpA expression plasmids (+) or vector controls (-) at different inducer concentrations; AHT for SosA and IPTG for CtpA. Cells were grown exponentially to an OD_600_ of 0.5, serially 10-fold diluted, and plated on TSA plus indicated inducer concentrations followed by incubation overnight at 37°C before imaging. (B) Severity of cell swelling by expression of SosA in a *ctpA* mutant is similar to expression of the stabilized truncated version SosAd10 in the wildtype. Evaluation of cell size distribution was performed by flow cytometry (FSC-A) of *S. aureus* JE2 and JE2-*ctpA* containing expression plasmids encoding full length SosA compared to the C-terminally truncated variant, SosAd10, expressed in the wildtype. Cells were grown exponentially prior to induction with 100 ng/ml of AHT and analyzed at indicated time points. Mean FSC-A values at indicated timepoints post induction are indicated as insert within histograms.

**Supplementary Figure S4.**

**
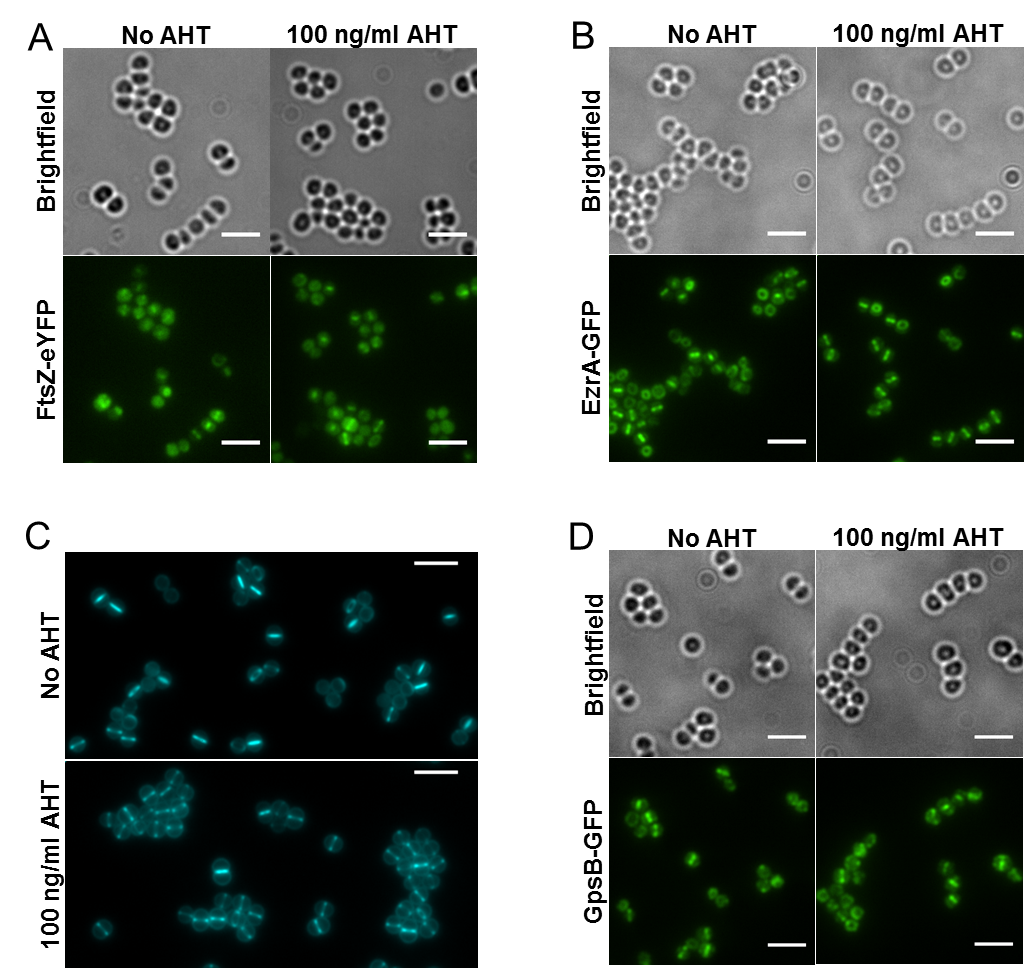
**

**Fig. S4** SosA halts septum completion. Localization of (A) FtsZ-eYFP in SJF4693 (JE2 pRAB12-*lacZ* pCQ11-FtsZ-eYFP ), (B) EzrA-GFP in SJF4696 (JE2 pRAB12-*lacZ* *ezrA-gfp*+) and (D) GpsB-GFP in SJF4699 (JE2 pRAB12-*lacZ* *gpsB-gfp*+) grown in the absence and presence of 100 ng/ml of AHT for 45 min. Fluorescence images are average intensity projections. Scale bars represents 3 µm. (C) Fluorescence microscopy images of JE2/pSosA grown in the absence or presence of 100 ng/ml of AHT for 45 min and labeled with HADA for 5 min. Images are average intensity projections. Scale bars represents 3 µm.

**Supplementary Figure S5.**


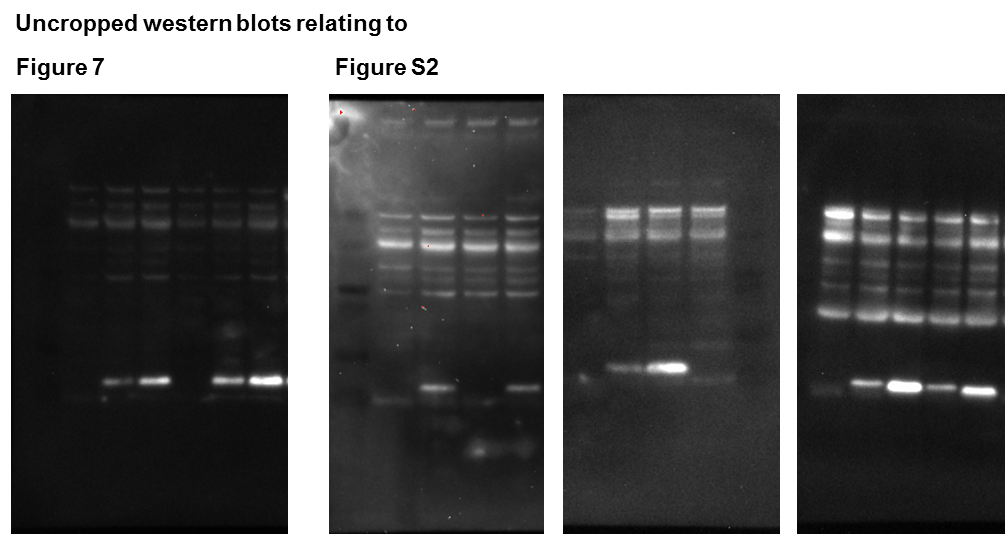


**Fig. S6** Uncropped western blots relating to Figure 7 and Figure S2.

**Supplementary Movie S1.**

Time-lapse microscopy of *S. aureus* JE2 WT and *sosA* mutant upon MMC exposure.

**Supplementary Movie S2.**

Time-lapse microscopy of *S. aureus* JE2 cells overexpressing SosA.

**Supplementary Movie S3.**

Time-lapse microscopy of *S. aureus* JE2 cells overexpressing SosAd10.

**References for Supplemental Material**

1. Monk IR, Shah IM, Xu M, Tan MW, Foster TJ. 2012. Transforming the untransformable: application of direct transformation to manipulate genetically Staphylococcus aureus and Staphylococcus epidermidis. MBio 3:e00277-11.
2. Monk IR, Tree JJ, Howden BP, Stinear TP, Foster TJ. 2015. Complete Bypass of Restriction Systems for Major Staphylococcus aureus Lineages. MBio 6:e00308-15.
3. Fey PD, Endres JL, Yajjala VK, Widhelm TJ, Boissy RJ, Bose JL, Bayles KW. 2013. A genetic resource for rapid and comprehensive phenotype screening of nonessential Staphylococcus aureus genes. MBio 4:e00537-12.
4. Chen J, Yoong P, Ram G, Torres VJ, Novick RP. 2014. Single-copy vectors for integration at the SaPI1 attachment site for Staphylococcus aureus. Plasmid 76:1-7.
5. Lund VA, Wacnik K, Turner RD, Cotterell BE, Walther CG, Fenn SJ, Grein F, Wollman AJ, Leake MC, Olivier N, Cadby A, Mesnage S, Jones S, Foster SJ. 2018. Molecular coordination of Staphylococcus aureus cell division. Elife 7:e32057.
6. Steele VR, Bottomley AL, Garcia-Lara J, Kasturiarachchi J, Foster SJ. 2011. Multiple essential roles for EzrA in cell division of Staphylococcus aureus. Mol Microbiol 80:542-555.
7. Helle L, Kull M, Mayer S, Marincola G, Zelder ME, Goerke C, Wolz C, Bertram R. 2011. Vectors for improved Tet repressor-dependent gradual gene induction or silencing in Staphylococcus aureus. Microbiology 157:3314-3323.
8. Brzoska AJ, Firth N. 2013. Two-plasmid vector system for independently controlled expression of green and red fluorescent fusion proteins in Staphylococcus aureus. Appl Environ Microbiol 79:3133-3136.
9. Bose JL, Fey PD, Bayles KW. 2013. Genetic tools to enhance the study of gene function and regulation in Staphylococcus aureus. Appl Environ Microbiol 79:2218-2224.
10. Karimova G, Ladant D. 2017. Defining Membrane Protein Topology Using pho-lac Reporter Fusions. Methods Mol Biol 1615:129-142.
